# The ancestral stringent response potentiator, DksA has been adapted throughout *Salmonella* evolution to orchestrate the expression of metabolic, motility, and virulence pathways

**DOI:** 10.1080/19490976.2021.1997294

**Published:** 2021-12-20

**Authors:** Helit Cohen, Boaz Adani, Emiliano Cohen, Bar Piscon, Shalhevet Azriel, Prerak Desai, Heike Bähre, Michael McClelland, Galia Rahav, Ohad Gal-Mor

**Affiliations:** a Sheba Medical Center, The Infectious Diseases Research Laboratory, Tel-Hashomer, Israel; b Sackler Faculty of Medicine, Tel Aviv University, Tel Aviv, Israel; c Department of Clinical Microbiology and Immunology, Tel Aviv University, Tel Aviv, Israel; d Janssen Research & Development, LLC, Raritan, New Jersey, USA; e Department of Microbiology and Molecular Genetics, University of California, Irvine, California, USA; f Hannover Medical School, Research Core Unit Metabolomics, Hannover, Germany

**Keywords:** *Salmonella*, DksA, virulence, pathogenicity, regulation, SPIs, HGT, RNA-Seq, enteric pathogens

## Abstract

DksA is a conserved RNA polymerase-binding protein known to play a key role in the stringent response of proteobacteria species, including many gastrointestinal pathogens. Here, we used RNA-sequencing of *Escherichia coli, Salmonella bongori* and *Salmonella enterica* serovar Typhimurium, together with phenotypic comparison to study changes in the DksA regulon, during *Salmonella* evolution. Comparative RNA-sequencing showed that under non-starved conditions, DksA controls the expression of 25%, 15%, and 20% of the *E. coli, S. bongori*, and *S. enterica* genes, respectively, indicating that DksA is a pleiotropic regulator, expanding its role beyond the canonical stringent response. We demonstrate that DksA is required for the growth of these three enteric bacteria species in minimal medium and controls the expression of the TCA cycle, glycolysis, pyrimidine biosynthesis, and quorum sensing. Interestingly, at multiple steps during *Salmonella* evolution, the type I fimbriae and various virulence genes encoded within SPIs 1, 2, 4, 5, and 11 have been transcriptionally integrated under the ancestral DksA regulon. Consequently, we show that DksA is necessary for host cells invasion by *S*. Typhimurium and *S. bongori* and for intracellular survival of *S*. Typhimurium in bone marrow-derived macrophages (BMDM). Moreover, we demonstrate regulatory inversion of the conserved motility-chemotaxis regulon by DksA, which acts as a negative regulator in *E. coli*, but activates this pathway in *S. bongori* and *S. enterica*. Overall, this study demonstrates the regulatory assimilation of multiple horizontally acquired virulence genes under the DksA regulon and provides new insights into the evolution of virulence genes regulation in *Salmonella spp.*

## Introduction

The ubiquitous bacterial genus *Salmonella* is a facultative intracellular animal and human pathogen, belonging to the Proteobacteria phylum. The *Salmonella* genus comprises the two defined species *Salmonella bongori* (*S. bongori*) and *Salmonella enterica* (*S. enterica*). *Salmonella* diverged from a common ancestor with *Escherichia coli* about 100–160 million years ago.^1,2^ The two current species of *Salmonella* diverged from a common ancestor about 40 to 63 million years ago.^1,3,4^ One of these species, *S. bongori*, is frequently associated with infection of cold-blooded animals, like reptiles and amphibians. The other species, *S. enterica*, includes seven distinct subspecies. Among the *S. enterica* subspecies known, subspecies *enterica* (ssp. I) is associated with human and warm-blooded animal infections and responsible for 99% of all human salmonellosis infections.^5^

Human infection by one of the *S. enterica* serovars can result in different clinical outcomes, ranging from asymptomatic infection, acute self‐limiting gastroenteritis, invasive systemic disease and bacteremia, to enteric (typhoid) fever. The result of a *Salmonella* infection is dependent on the nature of the infecting serovar and the immunological status of the host.^6^ These *S. enterica* infections are a significant cause of morbidity and mortality with an annual incidence of over 27 million cases of enteric fever^7^ and 78.7 million cases of gastroenteritis^8^ worldwide.

*Salmonella* speciation and the development of its pathogenicity have been fundamentally shaped by the horizontal acquisition of discrete genomic islands that encode new virulence capabilities including host cell invasion and intracellular pathogenesis.^3,9^ Many of the pan-*Salmonella* virulence genes are clustered within 23 horizontally acquired accessory genetic elements, known as *Salmonella* pathogenicity islands (SPIs).^10^ SPIs, like pathogenicity islands in other bacterial pathogens, are often located adjacent to tRNA genes and have been characterized as a “molecular toolbox” for bacterial virulence.^11^ The key acquisition of the *Salmonella* pathogenicity island 1 (SPI-1) prior to the divergence of *S. bongori* and *S. enterica* confers invasion ability into non-phagocytic host cells, via a type three secretion system (T3SS) and an associated array of secreted effectors encoded within and outside of SPI-1.^12,13^ *S. enterica* speciation was marked by the acquisition of SPI-2 that encodes a second T3SS, required for survival in professional phagocytes and long-term persistence in the host.^14,15^ Throughout *S. enterica* evolution, additional horizontally acquired SPIs have further shaped *Salmonella* virulence and host-specificity.

All living cells, including bacteria, need to sense and respond to nutrient deprivation and environmental stressors efficiently. This pathway, known as the “stringent response,” is primarily governed by the intracellular small signaling molecules guanosine-5′,3′-tetraphosphate, and pentaphosphate, collectively referred to as ppGpp.^16^ In proteobacteria species, the stringent response is also dependent on the RNA polymerase (RNAP)-binding protein, DksA that together with ppGpp, mediates a global transcriptional shift.^17^

DksA is a conserved 151–amino acids, 17.5 kDa transcription factor, containing a globular domain and a coiled coil structure that docks into the secondary channel of the β′ subunit of RNAP to influence RNAP activity.^18^ DksA binds RNAP either alone or in conjunction with ppGpp, which allosterically potentiates the function of DksA.^19^ The current view of how DksA regulates transcription suggests that the interaction of DksA with RNAP changes the stability of the open promoter complex, resulting in either a decrease or an increase in transcription initiation, depending on the properties of the target promoter.^20,21^ Other regulatory mechanisms of DksA may also include affecting transcription elongation,^22^ or fidelity^23^ of target genes.

Previously, we showed that in *S. enterica* serovar Typhimurium (*S*. Typhimurium), DksA is involved in the regulation of motility and biofilm formation, positively regulates SPI-1 genes, and is necessary for *S*. Typhimurium invasion into epithelial cells. In addition, we demonstrated that *dksA* is expressed during intestinal infection and is needed for gastrointestinal colonization and systemic infection in the mouse model.^24^ Nevertheless, it was unclear when during *Salmonella* evolution these roles have been acquired.

Here, we used comparative RNA-Sequencing (RNA-Seq) to systematically dissect the DksA regulon and identify the adaptation of the DksA regulatory circuits during *Salmonella* evolution, represented by *E. coli, S. bongori* and *S. enterica*. We found that in *Salmonella* spp., the DksA network was significantly rewired, inverted the directionality of the motility-chemotaxis regulation and evolved as a key coordinator of the type 1 fimbriae, SPI-1, and SPI-4 and of genes encoded on SPI-2, SPI-5, and SPI-11. In agreement with these findings, we show that DksA is required for growth in minimal medium by all three species, the invasion of *S*. Typhimurium and *S. bongori* into host cells and significantly contributes to the intracellular survival of *S*. Typhimurium in phagocytic cells.

## Results

### DksA controls metabolic and virulence pathways under non-stringent growth conditions

In recent years, several reports have shown that DksA may affect the transcription of target genes independently of ppGpp and plays a regulatory role beyond simply being an auxiliary factor of the stringent response.^25–27^ To systematically analyze DksA-dependent transcription and to follow its regulatory evolution, we applied comparative RNA-Seq and determined the transcriptome of the wild-type and its isogenic *dksA* mutant in *E. coli, S. bongori*, and *S*. Typhimurium strains, representing different phylogenetic branches during *Salmonella* evolution. Importantly, to determine what effect on transcription DksA has outside of the stringent response, all cultures were grown under non-starved conditions to late logarithmic phase in rich LB medium. Under these growth conditions, the cellular concentration of ppGpp is very low and was estimated to be in the range of only a few µM.^28^ Here, we were able to confirm the low abundance of ppGpp in *E. coli, S. bongori* and *S*. Typhimurium (between 50 to 250 fmol/ µg protein) and demonstrated similar concentrations in the wild-type and the *dksA* backgrounds, when cultures were grown to the late exponential phase in LB (Fig. S1). Bacterial RNA was harvested from three independent wild-type and Δ*dksA* mutant cultures of each species (18 cultures in total), and analyzed by deep RNA-Seq.

**Figure 1.**
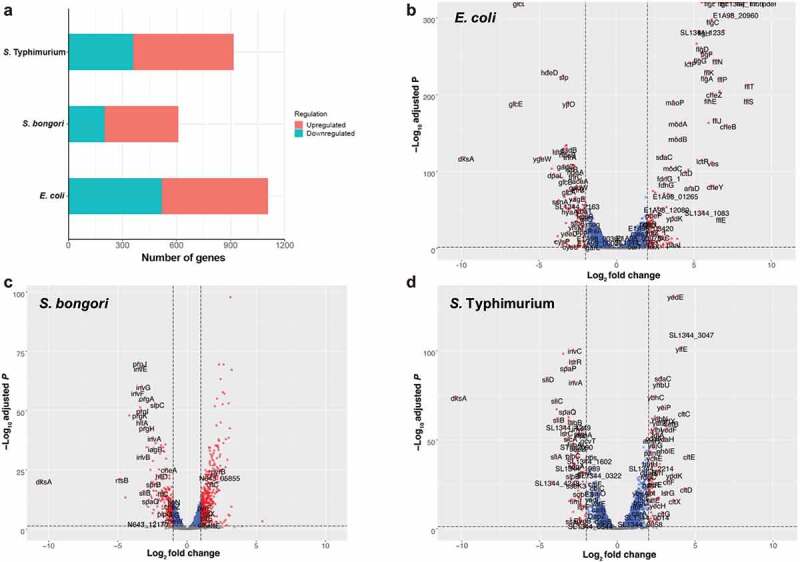
DksA is a pleiotropic regulator in *E. coli* and *Salmonella* spp. Comparative RNA-Seq analyses between a null *dksA* mutant and its isogenic wildtype strain were conducted in *E. coli, S. bongori*, and *S*. Typhimurium. (a) The number of DEGs, which were upregulated or downregulated by ≥ 2-fold in the *dksA* mutant relative to the wildtype background, is shown for each species. Volcano plots showing the fold change (log2 ratio) in the expression of genes in *ΔdksA* vs. the wildtype (X-axis) plotted against the -log_10_ adjusted *p*-value (Y-axis) in *E. coli* (b), *S. bongori* (c) and *S*. Typhimurium (d) are shown. Each dot on the plot represents the mean value (from three independent cultures) of one gene. Genes that were changed by more than 2-fold are colored in red. Orthologous genes were designated according to their names in *Salmonella.*

Overall, we identified 1106, 609, and 917 differentially expressed genes in the *ΔdksA* vs. the wildtype background that changed 2-fold or more (adjusted *P* < .05), corresponding to 25%, 15% and 20% of the *E. coli, S. bongori* and *S*. Typhimurium gene inventories, respectively (Figure 1(a)). In *E. coli*, 589 genes were upregulated, and 517 genes were downregulated (Figure 1(b); Table S1A); in *S. bongori*, 409 genes were upregulated, and 200 genes were downregulated (Figure 1(c); Table S1B); and in *S*. Typhimurium, 558 genes were upregulated, while 359 genes were downregulated (Figure 1(d); Table S1C) in the absence of DksA.

**Figure 2.**
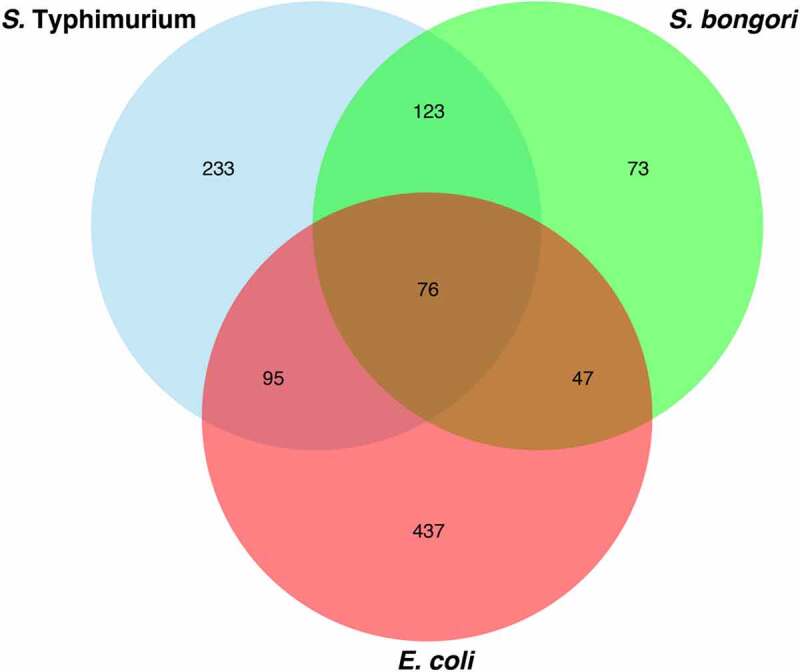
Core genes regulated by DksA in at least one species. The fold change in the expression of 2794 orthologous core genes present in *E. coli, S. bongori*, and *S*. Typhimurium was analyzed by RNA-seq. Venn diagram showing the number of common and species-specific DksA-regulated genes, whose expression was changed by ≥ 2-fold (adj *P* < .05)

To identify orthologous DksA-regulated genes in *S*. Typhimurium, *S. bongori*, and *E. coli*, we defined their core genome comprising 2,794 genes (Table S2A) and found that 76 genes were regulated by DksA in all of these three species. 20 genes are positively regulated by DksA in all three species, 50 genes are negatively regulated, and 6 genes are differentially regulated by DksA in *Salmonella* spp. and *E. coli*. In addition, 123 genes were commonly regulated by DksA in *S. bongori* and *S*. Typhimurium, 95 orthologous genes were DksA-regulated in *E. coli*, and *S*. Typhimurium, and 47 genes were mutually regulated by DksA in *E. coli* and *S. bongori* (Figure 2 and Table S2B). Importantly, these numbers do not include changes in the expression lower than 2-fold or an adjusted *P* > .05. In addition, the RNA-Seq workflow included an rRNA-depletion step, and so it did not identify any changes in rRNA gene expression.

Pathway enrichment analysis using the KEGG mapping tool demonstrated that common differentially expressed genes between the wild-type and *dksA* mutant in all three species were involved in metabolic pathways, biosynthesis of secondary metabolites, biosynthesis of amino acids, pyrimidine metabolism, quorum sensing, glycolysis, flagellar assembly and citrate (TCA) cycle (Fig. S2-S4).

### DksA regulates genes involved in citrate, glycogen and pyrimidine metabolism in *E.* *coli* and *Salmonella*

**Figure 3.**
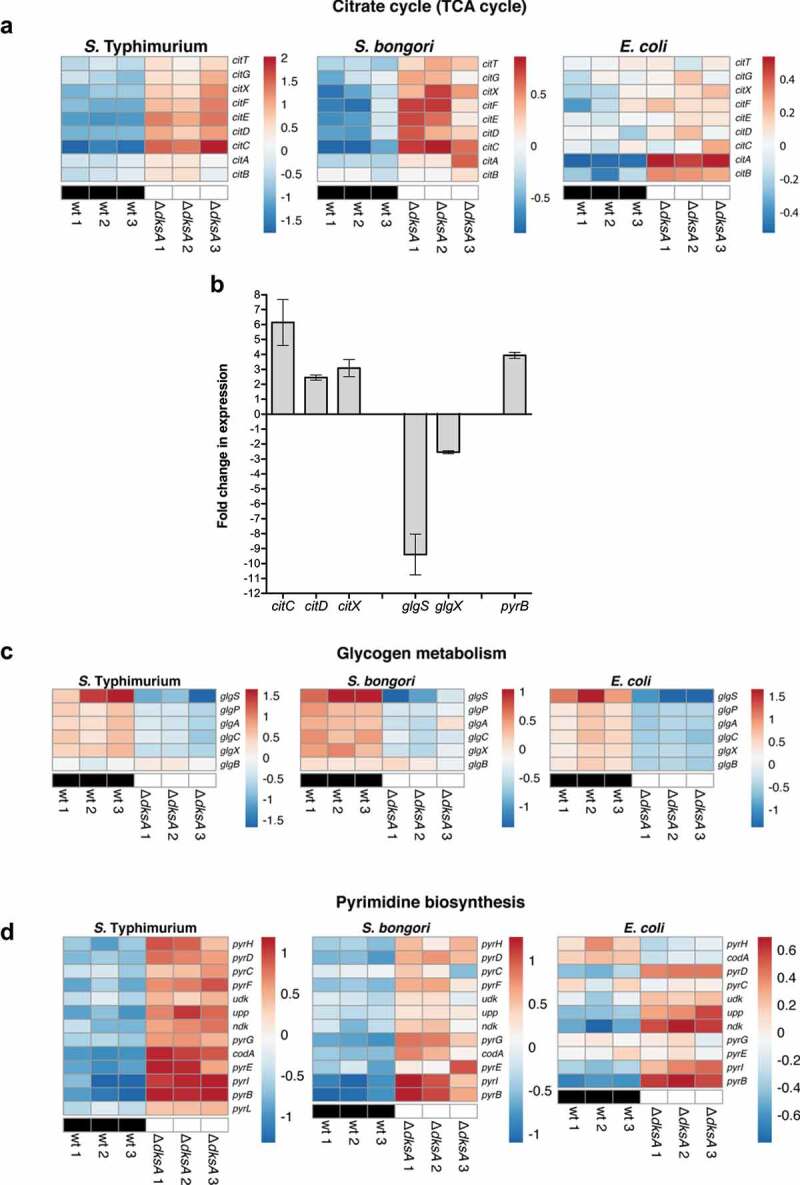
DksA regulates the citrate, glycogen, and pyrimidine metabolic pathways in *E. coli* and *Salmonella* spp. (a) Heat map based on RNA-Seq results showing the relative transcription of genes involved in the citrate (TCA) cycle. (b) The fold change in the expression of the citrate regulon (*citC, citD*, and *citX* genes), the glycogen regulon genes (*glgS* and *glgX*), and the pyrimidine biosynthesis gene (*pyrB*) in a *ΔdksA* relative to the expression in the wildtype strain was determined using qRT-PCR. RNA was extracted from late logarithmic phase *S*. Typhimurium cultures grown in LB broth at 37°C. The chart shows the mean value of 3–6 qRT-PCR reactions from two independent RNA extractions, and the error bars indicate the SEM. (c) Heat map based on RNA-Seq results showing the relative transcription of genes involved in glycogen metabolism and (d) pyrimidine biosynthesis

Some Enterobacteriaceae species, including *S. enterica*, are capable of utilizing citrate as a carbon and energy source. Under aerobic conditions, growth on citrate is dependent on an appropriate transport system and a functional tricarboxylic acid (TCA cycle, also known as Krebs or citric acid cycle). Citrate fermentation requires the functional citrate transporter CitT, the citrate lyase (encoded by *citCDEFXG*), and the two-component regulatory system encoded by *citAB*.^29^ RNA-Seq data showed that DksA strongly represses the citrate regulon in *S*. Typhimurium and also (although to a lesser extent) in *S. bongori* and *E. coli* (Figure 3(a)). qRT-PCR analysis confirmed these results and showed that in *S*. Typhimurium, in the absence of DksA, the expression of *citC, citD* and *citX* increased by 6, 2.5, and 3-fold, respectively (Figure 3(b)), indicating that DksA is a negative regulator of the citrate regulon in *S. enterica*.

Another important carbon metabolic pathway in Enterobacteriaceae is glycogen biosynthesis. Glycogen is a branched homopolysaccharide that accumulates in Enterobacteriaceae during growth arrest in the presence of a carbon source excess. In *Salmonella* and *E. coli*, glycogen is synthesized by both glycogen synthase GlgA and the branching enzyme GlgB. Glycogen biosynthesis is highly interlinked with a wide range of cellular and physiological processes and is dependent on the expression of the *glgBX, glgS*, and *glgCAP* operons.^30^ RNA-Seq analysis showed that expression of the entire glycogen biosynthesis regulon (*glgBXCAPS*) decreased in the absence of DksA in *E. coli, S. bongori*, and *S*. Typhimurium (Figure 3(c)). In agreement with these data, qRT-PCR experiments showed that *glgS* and *glgX* are downregulated by about 9 and 2.5-fold, respectively in the *dksA* background in *S*. Typhimurium (Figure 3(b)), indicating that DksA acts as an activator of the glycogen biosynthesis genes.

In contrast to glycogen biosynthesis, all of the pyrimidine biosynthesis genes (*pyrA, B, C, D, E, F, G, H, I* and *ndk*) were found to be significantly upregulated in *S*. Typhimurium and *S. bongori* and, to a lesser degree, in *E. coli* in the absence of DksA (Figure 3(d)). qRT-PCR analysis confirmed these results and showed that in an *S*. Typhimurium *dksA* background, the expression of *pyrB* is 4-fold higher than in the wild-type (Figure 3(b)), indicating that DksA conservatively represses the expression of the pyrimidine biosynthesis genes in *Salmonella* and *E. coli*.

Collectively, these results show a conserved transcriptional regulation of different metabolic pathways by DksA in *E. coli* and *Salmonella spp*, also under non-starved conditions.

### DksA is required for growth in minimal medium by *E.* *coli, S.* *bongori* and *S*. Typhimurium

**Figure 4.**
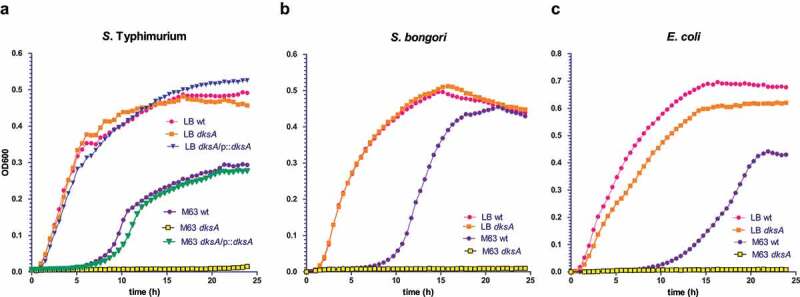
DksA is required for the growth of *E. coli, S. bongori* and *S*. Typhimurium in minimal medium. (a) The growth of *S*. Typhimurium SL1344, its derivative *dksA*-null mutant strain, and the *dksA* mutant harboring the *dksA* gene on pWSK29 was tested in LB or M63 minimal medium containing glycerol as a carbon source at 37°C, under aerobic growth conditions. The same analysis was performed with *S. bongori* strain SARC11 and its derivative *dksA*-null mutant strain (b) and with *E. coli* strain BW25113 and its derivative *dksA*-null mutant strain (c). Data present the mean optical density at 600 nm over time of triplicates in one representative experiment

Previously we showed that DksA is required for the growth of *S*. Typhimurium in minimal medium.^24^ The comparative RNA-seq results showing a common regulation of metabolic pathways and especially a conserved regulation of the TCA pathway, suggested that DksA may play a similar role also in *S. bongori* and *E. coli*. Therefore, the effect of DksA absence on bacterial growth was tested in all three species. These experiments showed that while DksA is not required for growth in rich medium, it was absolutely essential for growth of all of these species in minimal medium. Complementation of *dksA* from a low-copy plasmid in *S*. Typhimurium restored the ability of the ∆*dksA* strain to grow in minimal medium (Figure 4). These results indicated that DksA plays a conserved role as metabolic pathways regulator in both *E. coli* and *Salmonella spp*.

### DksA regulates the entire SPI-1 regulon in *Salmonella spp*

Many of the genes differentially expressed between the wild type and *dksA* mutant in both *S. bongori* (Fig. S3) and *S*. Typhimurium (Fig. S4), are involved in *Salmonella* infection and bacterial invasion of epithelial cells. This group includes virulence genes that are encoded in both *Salmonella* species, but absent in *E. coli* (e.g. SPI-1 genes) (Table S1D), or genes that are present in all three species, but are regulated by DksA only in *S. bongori* and *S. enterica* (e.g. type I fimbriae; see below).

**Figure 5.**
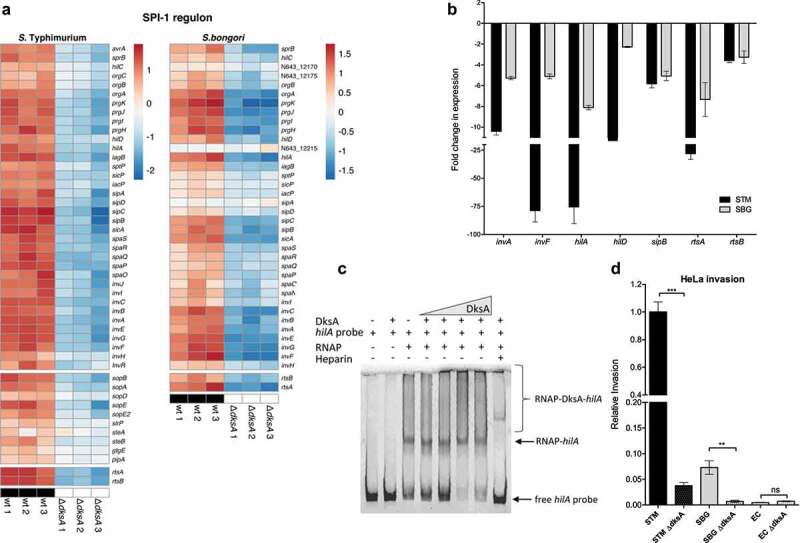
DksA activates the expression of SPI-1 genes in *Salmonella* spp. (a) Heat map of RNA-Seq results showing the relative transcription of SPI-1 genes (top block), SPI-1 effector genes encoded outside of SPI-1 (middle block), and the *rtsAB* SPI-1 related regulators (bottom block) in three independent wildtype and *ΔdksA S*. Typhimurium and *S. bongori* cultures. (b) The fold change in the expression of seven SPI-1 genes (*invA, invF, hilA, hilD, sipB, rtsA*, and *rtsB*) in a *dksA* mutant relative to the wildtype isogenic strain was determined using qRT-PCR. RNA was extracted from late logarithmic phase *S*. Typhimurium (STM), and *S. bongori* (SBG) cultures grown in LB broth at 37°C. The results show the mean value of 3–6 reactions from two independent RNA extractions, and the error bars indicate the SEM. (c) Gel mobility shift assay was performed by incubating 75 ng of a DNA probe corresponding to the regulatory region of *hilA* with increasing amounts of His-DksA (0.017 to 0.7 µg) and a constant amount of holoenzyme RNA-polymerase (1 unit). Heparin (2.5 µg) that prevents RNAP binding to DNA was added as a control to the reactions, as indicated. The DNA-protein complexes were resolved on 3.5% native acrylamide gel and stained using GelRed. (d) Bacterial invasion into epithelial HeLa cells was determined by the gentamicin protection assay. Invasion of all strains (relative to the *S*. Typhimurium WT background) was calculated as the ratio between the intracellular bacteria (CFU) recovered at 2 h p.i and the total number of CFU used to infect the cells. The mean of four independent infections with standard error of the mean are shown. *t*-test was used to determine statistical difference between the WT and the *dksA* backgrounds

SPI-1 is a 40 kb genomic island that was inserted into the ancient *Salmonella* genome upon divergence from *E. coli* about 160 million years ago, between the core genes *fhlA* and *mutS*, a known hotspot for the gain and loss of horizontally acquired genes.^31^ SPI-1 encodes the Type III secretion system (T3SS)-1 and a few of its associated effectors required for host cell invasion, intestinal colonization, and inflammation. Previously, we showed that DksA regulates some of the T3SS-1 genes and its associated effectors in *S*. Typhimurium and that in the absence of DksA, the invasion of *S*. Typhimurium into non-phagocytic cells is dramatically impaired.^24^ Here, we were able to extend our previous findings and show using RNA-Seq that in *S*. Typhimurium, all of the SPI-1 encoded genes, associated effectors encoded outside of SPI-1 genes (like *sopA, sopB, sopE, sopE2, steB*, and others) and its related regulatory system *rtsAB* are all downregulated in the absence of DksA. Moreover, similar results were also found in *S. bongori* (Figure 5(a)), indicating that SPI-1 regulation by DksA is conserved in both *Salmonella* species. qRT-PCR experiments have further confirmed these results and demonstrated that the expression of *invA, invF, hilA, hilD, sipB, rtsA*, and *rtsB* is significantly decreased in the *S*. Typhimurium and *S. bongori dksA* strains compared to the wildtype background. The most profound effect was found for *invF* and *hilA* that demonstrated more than a 75-fold decrease in expression in the absence of DksA in *S*. Typhimurium (Figure 5(b)).

These results raised the prospect of direct regulation of *hilA* by DksA. To further test the possibility of a DksA complex binding at this promoter, we applied electrophoretic mobility shift assay (EMSA) and tested the potential binding of the *hilA* promoter region in the presence of an *E. coli* RNA polymerase holoenzyme and His-DksA. As shown in Figure 5(c), we found that in the presence of RNAP and increasing quantities of His-DksA, the amount of unbound *hilA* probe decreased. The addition of heparin, which occupies the DNA binding sites on RNAP,^32^ eliminated most of the high molecular weight complexes and restored the appearance of the *hilA* unbound probe. These results support the possibility that DksA may bind at the *hilA* promoter region in conjunction with RNAP and suggest that the DksA regulation of SPI-1 genes is possibly mediated by the binding of an RNAP-DksA complex in the promoter region of the SPI-1 master regulator gene, *hilA*.

Since SPI-1 expression is absolutely required for *Salmonella* invasion into host cells, we next compared the contribution of DksA to *Salmonell*a invasion into non-phagocytic cells. Previously we showed its role in *S*. Typhimurium,^24^ but since our current RNA-seq analysis suggested a conserved role of DksA in SPI-1 expression across both *Salmonella* species, we seek out to test its effect also in *S. bongori*. As seen in Figure 5(d), and in agreement with the RNA-seq data, the absence of DksA significantly attenuated the ability of both *Salmonella* species to invade HeLa cells. These results indicate that the assimilation of SPI-1 under the DksA regulatory umbrella is common to both *S. enterica* and *S. bongori* and most likely evolved in their last common ancestor.

### DksA regulates several SPI-2 genes under SPI-1 inducing conditions

**Figure 6.**
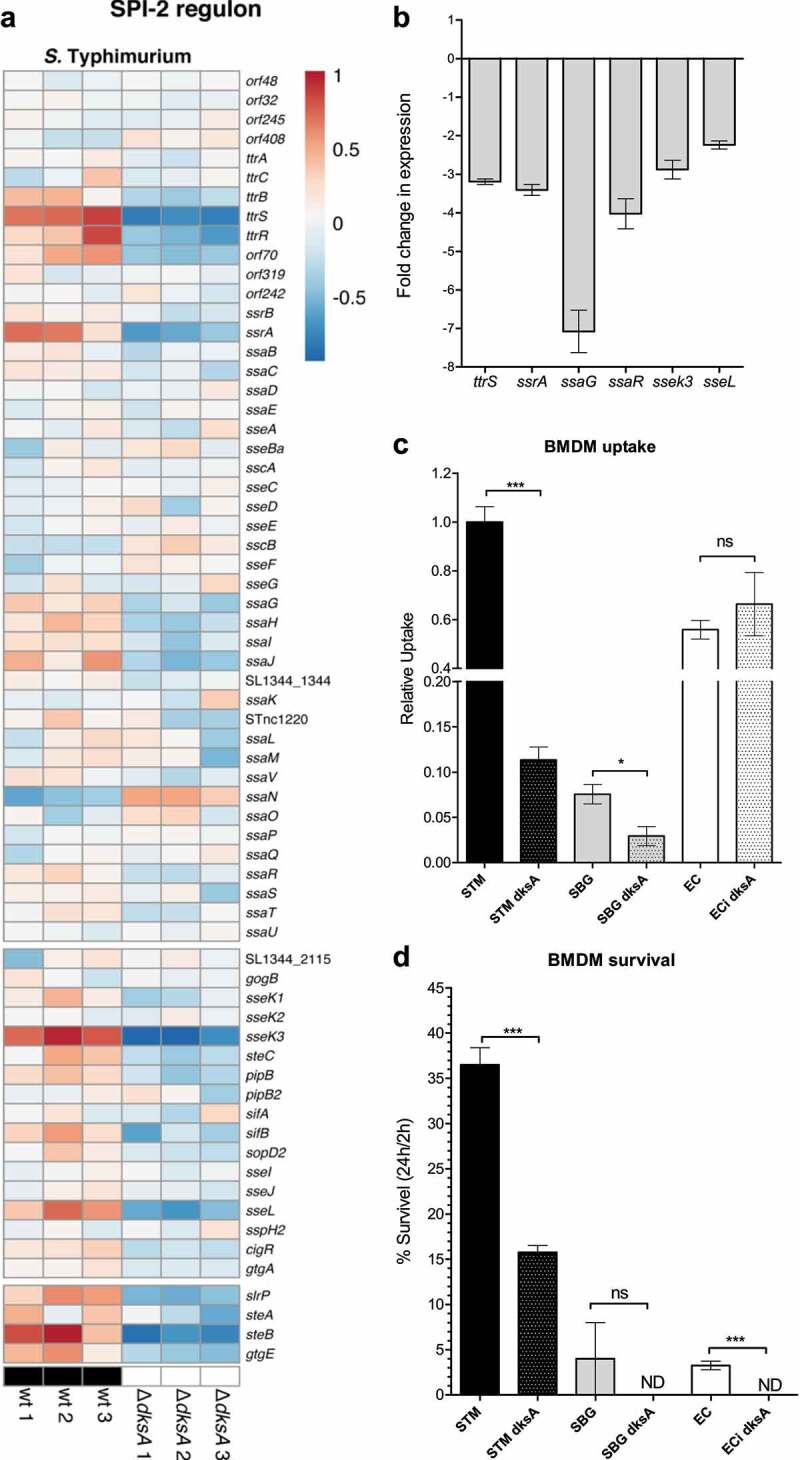
DksA regulates several SPI-2 genes under SPI-1-induced conditions. (a) Heat map of RNA-Seq results showing the relative transcription of SPI-2 genes (top block), SPI-2 effector genes encoded outside of SPI-2 (middle block) and effectors known to be translocated by both T3SS-1 and T3SS-2 (bottom block) in three independent wildtype and Δ*dksA S*. Typhimurium cultures, grown to late logarithmic phase in LB (considered as SPI-1 induced conditions). (b) The fold change in the expression of six SPI-2 genes (*ttrS, ssrA, ssaG, ssaR, sseK3*, and *sseL*) in a *dksA* mutant relative to the wildtype isogenic strain was determined using qRT-PCR. RNA was extracted from late logarithmic phase *S*. Typhimurium cultures grown in LB broth at 37°C. The results show the mean value of 3–6 reactions from two independent RNA extractions, and the error bars indicate the SEM. (c) Infection of BMDMs was determined by the gentamicin protection assay and is presented relative to *S*. Typhimurium SL1344. Uptake was calculated as the ratio between the intracellular bacteria (CFU) recovered at 2 h p.i and the total number of CFU used to infect the cells. (d) Bacterial survival was determined as the ratio between the intracellular bacterial load (CFU) at 24 h p.i and the number of recovered CFUs at 2 h p.i. The mean of four independent infections with standard error of the mean are shown. *t*-test was used to calculate the statistical difference between the WT and the *dksA* backgrounds

SPI-2 is 40 kb, integrated at the tDNA-Val locus, and contains a 25 kb segment encoding the T3SS-2 and a 15 kb section encoding the tetrathionate reductase gene clusters (*ttr*ABCRS), involved in anaerobic respiration.^31^ In contrast to the uniform regulation of SPI-1 by DksA, RNA-Seq showed that in *S*. Typhimurium, only a handful of SPI-2 genes were regulated by DksA under the experimental setting (late logarithmic growth phase in LB), considered as SPI-1 inducing conditions. The genes *ttrB, ttrS, ttrR* and *orf70* encoded within the tetrathionate locus, the regulatory gene *ssrA*, two SPI-2 effector genes (*sseK3* and *sseL*), and four dual T3SS-1 and T3SS-2 translocated effector encoded genes (*slrP, steA, steB*, and *gtgE*) were significantly repressed in *S*. Typhimurium lacking DksA. Similarly, a moderate decrease in the expression of *ssaG, ssaH, ssaI*, and *ssaJ*, was identified under these conditions (Figure 6(a)). qRT-PCR analysis confirmed the RNA-Seq results and demonstrated 2 to 7-fold decrease in the expression of *ttrS, ssrA, ssaG, ssaR, sseK3*, and *sseL* (Figure 6(b)).

These results suggested that since DksA is involved in the regulation of at least a few SPI-2 genes, it may contribute to the intracellular survival of *Salmonella*. To test this possibility, the uptake and survival of the studied three species and their *dksA* mutant strains were used to infect bone marrow-derived macrophages (BMDMs). The uptake of both *S*. Typhimurium and *S. bongori dksA* mutants was reduced relative to their WT strains (Figure 6(c)), probably due to the contribution of T3SS-1 to *Salmonella* entry, even into phagocytic cells. In addition, a *S*. Typhimurium *dksA* mutant strain was significantly impaired in intracellular survival compared to the WT background (Figure 6(d)). These results are in agreement with a previous report by Henard and colleagues^33^ and indicate that DksA is required for intracellular survival of *S. enterica* in macrophages, at least partly, due to its role in SPI-2 regulation. Noteworthy, the intracellular survival of *S. bongori* and *E. coli* in BMDM was very low to begin with (since they don’t carry the SPI-2), however the lack of DksA in these species, even further reduced their viability to an undetected level. These results suggest additional intracellular role for DksA, which is SPI-2-independent, such as its previously reported role in *S*. Typhimurium tolerance to reactive oxygen and nitrogen species.^33–35^

### DksA regulates SPIs 4, 5 and 11

**Figure 7.**
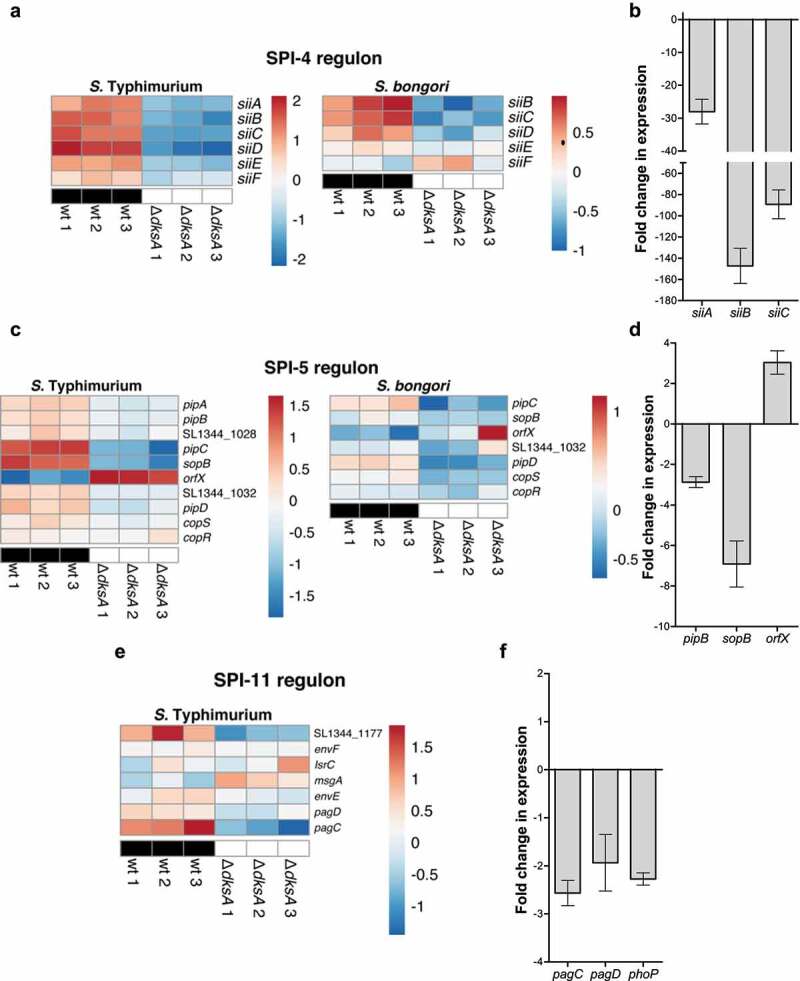
DksA regulates the expression of SPI-4, 5 and 11 genes in *Salmonella* spp. (a) Heat map of RNA-Seq results showing the transcription of six SPI-4 genes in three independent wildtype and *ΔdksA S*. Typhimurium and *S. bongori* cultures. (b) The fold change in the expression of three SPI-4 genes (*siiA, siiB*, and *siiC*) in a *dksA* mutant relative to the wildtype isogenic strain was determined using qRT-PCR. RNA was extracted from late logarithmic phase *S*. Typhimurium cultures grown in LB broth at 37°C. The chart bars show the mean value of 3–6 reactions from two independent RNA extractions ± SEM. (c) Heat map of RNA-Seq results displaying the transcription of SPI-5 genes in three independent wildtype and *ΔdksA S*. Typhimurium and *S. bongori* cultures. (d) The fold change in the expression of three SPI-5 genes (*pipB, sopB*, and *orfX*) in a *dksA* mutant relative to the wildtype isogenic strain was determined using qRT-PCR. RNA was extracted from late logarithmic phase *S*. Typhimurium cultures grown in LB broth at 37°C. (e) Heat map of RNA-Seq results showing the transcription of seven SPI-11 genes in three independent wildtype and *ΔdksA S*. Typhimurium cultures. (f) The fold change in the expression of *pagC, pagD*, and *phoP* in a *dksA* mutant relative to the wildtype isogenic strain was determined using qRT-PCR. RNA was extracted as above

SPI-4 spans a 27 kb segment that was inserted adjacent to the tRNA-like gene *ssb*.^36^ SPI-4 encodes a Type 1 secretion system and the major adhesin SiiE, required for intestinal epithelial cells attachment and invasion.^37^ Like SPI-1, the expression of SPI-4 genes was found by RNA-Seq to be significantly downregulated in the *dksA* background in both *S*. Typhimurium and *S. bongori* (Figure 7(a)). qRT-PCR further confirmed these results and showed a 28 to 147-fold decrease in the expression of *siiA, siiB*, and *siiC* in the *S*. Typhimurium *dksA* mutant compared to the wild type strain (Figure 7b).

SPI-5 corresponds to a small 7.6 kb island integrated adjacent to the tDNA-Ser (rna 42). SPI-5 encodes the T3SS-1 effector SopB that manipulates the host Rho-GTPase and is required for *Salmonella* invasion, membrane ruffling, and activation of pro-inflammatory response.^38^ In addition, SPI-5 harbors the T3SS-2 effector PipB that contributes to intra-macrophage survival.^39^ SPI-5 has a mosaic structure, and in *S. bongori*, only part of the SPI-5 genes are present, including *sopB*, but excluding *pipB*. In *S*. Typhimurium, most of the SPI-5 genes and especially *pipC* and *sopB*, were downregulated in the absence of DksA. At the same time, *orfX* demonstrated an opposite transcriptional pattern and was upregulated in the *dksA* background. Similar, but less pronounced trend in expression of SPI-5 genes (especially *pipC* and *pipD*), were also seen in *S. bongori* (Figure 7(c)). qRT-PCR confirmed these results and showed a 3- and a 7-fold decrease in the expression of *pipB* and *sopB*, respectively, and a 3-fold increase in the transcription of *orfX* in the absence of DksA in *S*. Typhimurium (Figure 7(d)).

SPI-11 encodes several genes, including *pagC, pagD*, and *msgA* belonging to the PhoPQ regulon, which is essential for *Salmonella* virulence and intracellular replication within macrophages.^40^ In *S*. Typhimurium, both *pagC, pagD*, and *phoP* were found by RNA-Seq to be expressed at moderately lower levels in the absence of DksA (Figure 7(e)) and qRT-PCR (Figure 7(f)).

Collectively, these results indicated that multiple virulence genes and diverse pathogenicity islands that were acquired horizontally during *Salmonella* evolution were inserted under the preexisting DksA regulon.

### DksA regulates the type I fimbria in *S*. Typhimurium but not in *E.* *coli*

**Figure 8.**
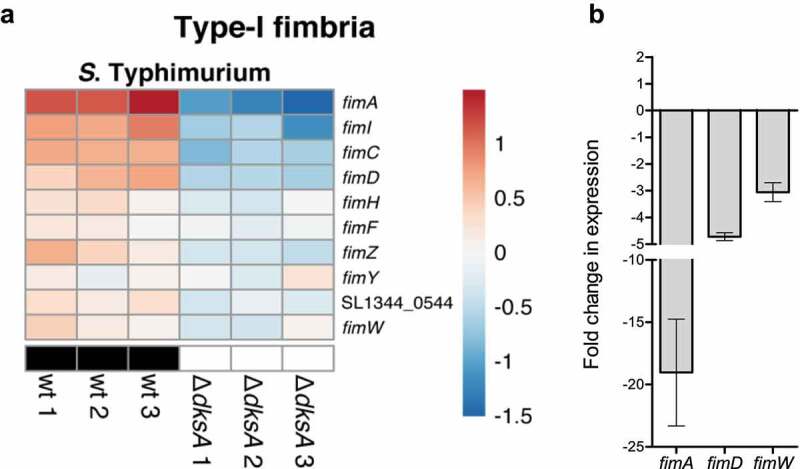
DksA activates the type-I fimbria genes in *S*. Typhimurium. (a) Heat map of RNA-Seq results displaying the transcription of 10 type-I fimbria genes in three independent wildtype and *ΔdksA S*. Typhimurium cultures. (b) The fold change in the expression of *fimA, fimD*, and *fimW* genes in a *dksA* mutant relative to the wildtype isogenic strain was determined using qRT-PCR. RNA was extracted from late logarithmic phase *S*. Typhimurium cultures grown in LB broth at 37°C

Type 1 fimbriae are known for their ability to mediate mannose-sensitive adhesion to host cells and are common colonization factors expressed by members of the Enterobacteriaceae family.^41^ In *E. coli*, type 1 fimbriae are encoded by the *fim* cluster composed of a polycistronic operon of the seven structural genes (*fimAICDFGH*) and two monocistronic operons encoding the site-specific recombinases, FimB and FimE.^42^ In *S*. Typhimurium, *fimA* expression is regulated transcriptionally by the adjacent regulatory proteins FimZ and FimY and by the relative concentrations of the rare tRNA-Arg (UCU), located adjacent to the *fim* cluster encoded by *argU* (*fimU*).^43^ Our RNA-Seq analysis clearly showed that in *S*. Typhimurium (Figure 8(a)), but not in *E. coli* (data not shown), the *fim* regulon is positively regulated by DksA, and in the absence of DksA its expression is significantly decreased. Confirmatory qRT-PCR experiments further supported these results and showed that in *S*. Typhimurium, the expression of *fimA, fimD*, and *fimW* is reduced by 19, 5, and 3-fold, respectively, in the *dksA* mutant strain, compared to the wild-type (Figure 8(b)). Hence, these results indicate rewiring of the *fim* regulation between *E. coli* and *Salmonella*, while integrating the type I fimbriae genes transcription to the DksA regulon in *S*. Typhimurium.

### DksA controls the lsr autoinducer-2 regulon in *S*. Typhimurium and in *E.* *coli*

**Figure 9.**
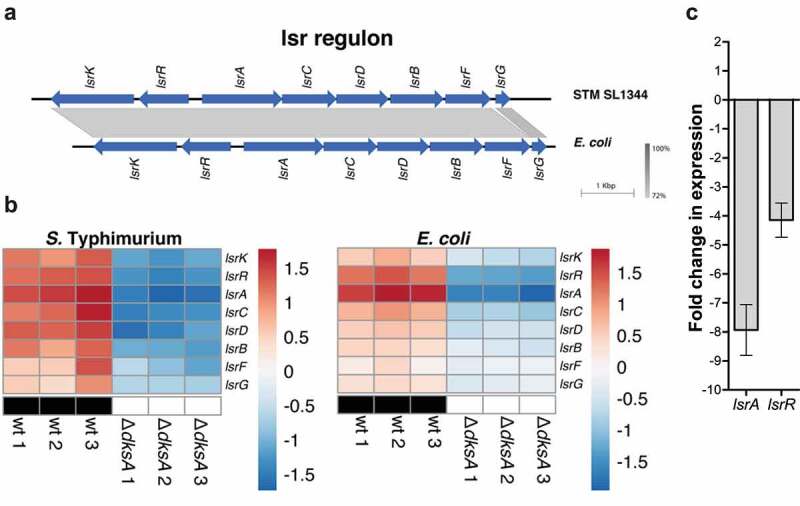
DksA activates the *lsr* autoinducer-2 regulon in *E. coli* and *S*. Typhimurium. (a) Genetic organization of the *lsr* locus in *S*. Typhimurium (STM) SL1344 (GenBank: FQ312003.1; position 4,302,044 to 4,311,611) and *E. coli* BW25113 (GenBank: CP009273.1; position 1,592,536 to 1,601,208) is shown. The gray regions indicate the degree of DNA similarity. (b) Heat map of RNA-Seq results showing the transcription of eight *lsr* genes in three independent wildtype and *ΔdksA S*. Typhimurium and *E. coli* cultures. (c) The fold change in the expression of *lsrA and lsrR* genes in a *dksA* mutant relative to the wildtype isogenic strain was determined using qRT-PCR. RNA was extracted from late logarithmic phase *S*. Typhimurium cultures grown in LB broth at 37°C

Population-dependent behavior is mediated in many bacteria species by a process known as quorum sensing. Global changes in gene expression in response to quorum sensing are directed by the synthesis, release, and detection of small signal molecules termed autoinducers. Different bacterial phenotypes are affected by quorum sensing, including motility, biofilm formation, bioluminescence, and virulence.^44^ Autoinducer-2 (AI-2) is a universal signal molecule that could be synthesized and recognized by many Gram-positive and Gram-negative bacteria and plays a role in interspecies communication.^45^ In *E. coli* and *S*. Typhimurium, AI-2 is imported from the environment into the cell by the Lsr transporter system, encoded the genes *lsrA, lsrB, lsrC, lsrD, lsrF*, and *lsr*G. In addition, the adjacent and divergently transcribed genes *lsrR* and *lsrK* are responsible for the regulation of the *lsr* operon (Figure 9(a)).^46^ Interestingly, RNA-Seq data showed that the entire *lsr* regulon was downregulated in the absence of DksA in *S*. Typhimurium and in *E. coli* (Figure 9(b)). qRT-PCR analysis further confirmed these results and showed a decrease of 8- and 4-fold in the expression of *lsrA* (that encodes an ATP-binding protein for the AI-2 ABC transporter complex) and *lsrR* (encoding for a transcriptional regulator of the *lsr* operon), respectively, in the *S*. Typhimurium *dksA* mutant strain in comparison to the wildtype (Figure 9(c)). These results show for the first time that the universal quorum-sensing autoinducer AI-2 system in *Salmonella* and in *E. coli* is under the positive regulation of DksA.

### DksA regulates the motility-chemotaxis regulon in *E.* *coli* and *Salmonella spp*. in an opposite directionality

In *E. coli* and *Salmonella*, more than 50 genes are involved in the formation and function of the flagella, and their assembly is tightly controlled by a highly organized and hierarchical regulatory cascade. The flagellar, motility, and chemotaxis genes are all organized under one regulon that composes three ordered classes corresponding to classes I (early), II (middle), and III (late) regulatory phases.^47^

**Figure 10.**
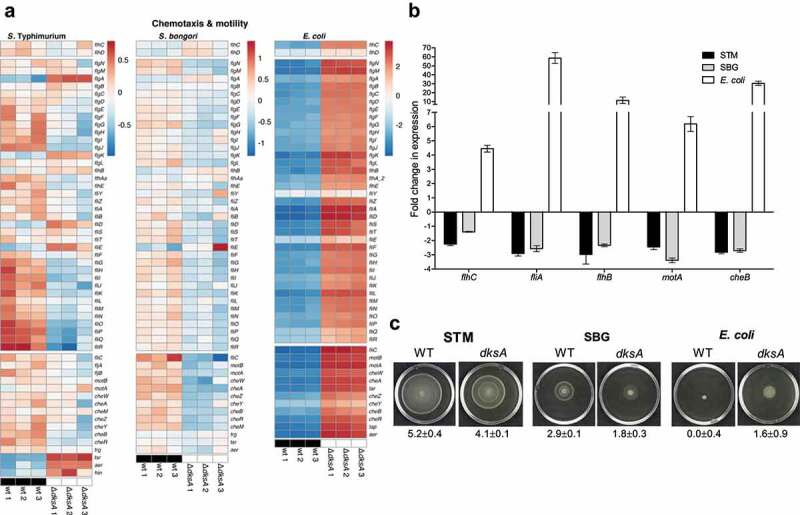
DksA regulates the motility-chemotaxis regulon in *E. coli* and *Salmonella* spp. in an opposite manner. (a) Heat map based on RNA-Seq results showing the transcription of the motility-chemotaxis regulon in three independent wildtype and *ΔdksA* cultures of *S*. Typhimurium, *S. bongori* and *E. coli*. Class I, II, and III genes are shown in the top, middle, and bottom blocks, respectively. (b) The fold change in the expression of *flhC* (class I), *fliA* and *flhB* (Class II), and *CheB* and *motA* (Class III) in a *dksA* mutant relative to the expression in the wildtype isogenic strain was determined using qRT-PCR. RNA was extracted from the late logarithmic phase of *S*. Typhimurium (STM), *S. bongori* (SBG), and *E. coli* cultures grown in LB broth at 37°C. (c) Motility of wildtype and *ΔdksA* strains of *S*. Typhimurium, *S. bongori* and *E. coli* was examined on soft agar plates for 5 h at 37°C. An image of one representative plate is shown. The mean motility value (in cm) and the standard deviation (of five independent plates) are shown underneath each image

The RNA-Seq analysis demonstrated that the flagella-chemotaxis regulon in *E. coli* is repressed by DksA, as in the *dksA* mutant all the genes besides *fliY* (i.e. 50 out of 51 genes of this regulon) were upregulated (Figure 10(a)). Nonetheless, the expression of these genes in *S. bongori* and *S*. Typhimurium was oppositely regulated by DksA and the expression of many of them decreased in the absence of DksA, indicating that in the *Salmonella* species, they are under a positive regulation of DksA. An exception for this regulatory pattern in *S*. Typhimurium was observed for only a few genes in the regulon including *flgA, flgK, tsr, aer*, and *hin*. An independent qRT-PCR approach confirmed these results and demonstrated that *flhC* (Class I), *fliA* and *flhB* (class II), *motA*, and *cheB* (Class III) are downregulated in *S*. Typhimurium and *S. bongori*, but upregulated in *E. coli* in the absence of DksA (Figure 10(b)). In agreement with the expression results, motility assays on soft agar plates revealed that a *dksA* null deletion results in a moderate (20–40%), but statistically significant reduction in the motility of *S*. Typhimurium and *S. bongori*, but increased *E. coli* motility, in comparison to the wild-type strains (Figure 10(c) and S5). These data are consistent with an earlier report showing upregulation of genes involved in flagellum synthesis in a *dksA E. coli* K-12 strain MG1655,^48^ and together indicate that during *Salmonella* speciation the directionality of the motility-chemotaxis regulation by DksA has been flipped and changed from a negative regulator in *E. coli* to an activator in *Salmonella spp*.

In Summary, our results show that DksA is a pleiotropic regulator that conservatively controls multiple ancestral metabolic and physiological pathways in *E. coli* and in both *Salmonella* species. Throughout *Salmonella* evolution, rewiring of specific common pathways occurred and several key virulence pathways, which were horizontally acquired into the *Salmonella* genome, were inserted under the regulatory umbrella of DksA. As a result, DksA has maintained it role as a major metabolic coordinator, but further evolved as a key virulence *Salmonella* regulator.

## Discussion

Bacterial pathogens have adapted to utilize distinct metabolic and virulence pathways allowing them to colonize and compete with the microbiota within their host. Many of these fitness-specific traits have been gained via horizontal gene transfer or changed their ancestral function during evolution of a pathogenic lifestyle. Since these virulence factors are often carried on xenogeneic DNA elements, they must be appropriately assimilated in the preexisting regulatory network of their bacterial carrier, to be expressed at the required time and place during host infection and transmission, without causing intolerable metabolic burden.^49^ Here, we used deep RNA-sequencing to investigate the adaptation of the DksA regulatory circuit during *S. enterica* evolution, from a nonpathogenic ancestor to a pathogen of warm-blooded animals.

RNA-Seq analysis of three bacterial species representing this evolutionary course demonstrated that under non-starved conditions, DksA affects a significant portion (15–25%) of the *E. coli, S. bongori*, and *S*. Typhimurium genes, indicating that DksA plays a key role as a transcriptional coordinator that goes beyond its role as a stringent response potentiator. DskA regulates a similar set of metabolic and physiological pathways in all three species. These pathways include biosynthesis of amino acids, secondary metabolites, and sulfur metabolism, the TCA cycle, glycogen, pyrimidine biosynthesis and tRNA genes. Moreover, we showed that DksA is a positive regulator of quorum sensing and controls the expression of genes required for the production of the universal bacterial communication molecule autoinducer-2. Finally, we provide genome-scale evidence that during *Salmonella* evolution, multiple virulence pathways have been transcriptionally integrated into the DksA regulon.

DksA was previously shown to regulate pathogenicity and virulence-associated phenotypes in multiple Gram-negative animal and plant pathogens, including the expression of the T3SS in *Erwinia amylovora*^50,51^ and the growth of *Xanthomonas citri* in its host plants.^52^ In *Pseudomonas aeruginosa*, DksA was reported to coordinate quorum sensing, anaerobiosis, and motility^53,54^ and to affect the production of the cholera toxin and hemagglutinin protease in *Vibrio cholera*.^55,56^ In *Haemophilus ducreyi*, DksA was also reported to regulate pathogenicity,^57^ and the expression of T4SS in *Bartonella henselae*.^58^ In *Legionella pneumophila*, DksA was shown to be involved in stationary-phase survival, flagellar gene activation, lysosome avoidance, and macrophage cytotoxicity,^59^ and in *Campylobacter jejuni*, a *dksA* mutant strain was impaired in invading intestinal cells and manipulating the host immune response.^60^ Furthermore, DksA was demonstrated to control the expression of the genes in the LEE pathogenicity island in Enterohaemorrhagic *E. coli*^61^ and to be involved in virulence and intercellular spread of *Shigella flexneri*.^62^ Finally, in *S*. Typhimurium, DksA was reported to play a role in resistance to reactive oxygen and nitrogen species,^33,35^ and a *dksA* mutant strain was significantly impaired in host cells invasion^24^ and attenuated in J774A.1 macrophage-like cells,^63^ chicken^64^ and mouse^24,65^ infection models.

Łyżeń and colleagues have previously demonstrated that in the presence of ppGpp, DksA strongly represses the transcription of the *argX* promoter, which controls the expression of the four tRNA genes *argX, hisR, leuT*, and *proM*. However, in the absence of ppGpp, under non-starved rapid cellular growth, DksA can activate transcription from the *argX* promoter.^25^ These results suggested that DksA might contribute to tRNA transcription regulation in *E. coli* either as a positive or negative regulator. Here, we showed that in *S*. Typhimurium, many of the genes organized in SPI-2, SPI-4, SPI-5, and in the *fim* cluster, which are all integrated into tDNA or tDNA-like sites are under the regulation of DksA. These results may suggest that the ancestral regulation of tRNA genes by DksA has evolved to control the expression of pathogenicity island genes, after their integration into tRNA loci.

Besides metabolic pathways, quorum sensing, and pathogenicity, another phenotype found to be under the DksA control is the motility and chemotaxis of *E. coli, S. bongori*, and *S. enterica*. We showed that, while in *E. coli* DksA acts as a repressor of this regulon, the regulatory activity of DksA has been inverted in *S. bongori* and *S*. Typhimurium, in which DksA positively regulates many of the motility-chemotaxis genes. Previously, it was suggested that the directionality of DksA regulation is dependent on the precise promoter architecture of its target genes.^20,21^ Here, we hypothesize that the observed change in motility regulation by DksA between *E. coli* and *Salmonella* has evolved to better synchronize flagellum-based motility and invasion in *Salmonella* spp. Like SPI-1- and SPI-4-dependent invasion, *Salmonella* motility is largely required for host colonization and full virulence.^66,67^ Previously, we demonstrated that *dksA* is readily expressed within the host during intestinal colonization,^24^ and a tight regulatory linkage between invasion and motility in *Salmonella* has been established by others.^68,69^ Further studies have also demonstrated that flagellum-dependent motility drives *Salmonella* into a near-surface swimming mode that facilitates its invasion by scanning the surface of host cells and the selection of permissive entry sites.^70,71^ Our current results propose that DksA is an additional regulatory tool used by *Salmonella* to synchronize motility and invasion into host cells by co-activation of SPI-1, SPI-4, assorted SPIs-2, 5, and 11 encoded genes, type I fimbria and motility.

Changes in the DksA regulatory setup may have evolved by adaptation of newly acquired promoters to facilitate direct binding of an RNAP-DksA complex. For example, our results support the possibility that a DksA-RNAP complex binds at the regulatory region of *hilA*, the SPI-1 master regulator, and by that mechanism may affect the transcription of the entire SPI-1 regulon. Nonetheless, additional experimental approaches, such as *in-vitro* transcription, are needed to further confirm this possibility. Another indirect mechanism responsible for changes in the DksA regulon might be changes in the expression pattern of other global regulators, downstream to DksA. For example, we found that the lack DksA, leads to a significant increase in the expression of the flagellar sigma factor *fliA* (sigma 28) in *E. coli*, but not in *Salmonella spp*. (Figure 10 and Fig. S7A), explaining the increased expression of motility and chemotaxis genes in this species. In contrast, the lack of DksA resulted in a very prominent decrease in the expression of the stress response sigma factor, RpoS (sigma 38) in *E. coli*, while the expression of RpoS was not significantly affected in *Salmonella* Typhimurium grown to the late logarithmic phase (Fig. S7). Furthermore, the expression of the alternative sigma factor *rpoE* (sigma 24) was also found to significantly decrease in the absence of DksA in both *Salmonella* species, but not in *E. coli* (Fig. S7A). Therefore, these differences can contribute to changes in the expression of RpoE- and RpoS-regulated genes in an indirect DksA-dependent manner.

To summarize, RNA-Seq combined with additional molecular approaches and phenotypic studies have demonstrated that DksA plays a pleiotropic role in controlling the transcription of numerous physiological and metabolic pathways. The regulation of these pathways by DksA is not limited to starvation and stress conditions as is currently accepted, but also occurs under non-starved growth conditions, in the presence of low concentrations of ppGpp. During *Salmonella* evolution and its adaptation from an environmental bacterium to a pathogen, DksA has evolved by regulatory rewiring to a key pathogenicity regulator that controls the expression of discrete master regulators and virulence gene clusters that were inserted into tDNA loci. This mechanism allows *Salmonella* to orchestrate the expression of a large number of horizontally acquired genes by a preexisting transcription factor, and synchronize metabolic, motility, quorum sensing and virulence phenotypes required for efficient infection and transmission in tandem. We suggest that the role of DksA as a global virulence coordinator is not unique to *Salmonella* and it functions by a similar mechanism in other Gram-negative pathogens that carry horizontally acquired virulence genes.

## Materials and methods

**Bacterial strains and growth conditions**. Bacterial strains and plasmids utilized in this study are listed in Table S3A. *S*. Typhimurium SL1344, *S. bongori* NCTC 12419 (also known as Salmonella Reference Collection C strain SARC11) and *E. coli* BW25113, and their isogenic *dksA* null mutant strains were routinely grown at 37°C in Lennox Luria-Bertani (LB; BD Difco) or in M63 minimal medium under aerobic conditions. When needed, 100 µg/ml ampicillin, 50 µg/ml kanamycin, and 25 µg/ml chloramphenicol were added to the media.

**Cloning and mutants construction**. All primers used in this study are listed in Table S3B. Oligonucleotides were purchased from IDT, and PCR was carried out using Phusion Hot Start Flex DNA Polymerase (Thermo). In-frame deletion of *dksA* in *S. bongori* SARC11 was constructed by the λ-red-recombination system in a three steps PCR method to produce an amplimer containing the Chloramphenicol resistance gene, as described in.^72^ The resistance cassette was then eliminated from the genome by using a helper plasmid (pCP20) carrying the FLP recombinase.^73^

**RNA Sequencing**. Three independent cultures of wildtype and Δ*dksA* strains of *S*. Typhimurium SL1344, *S. bongori* NCTC 12419, and *E. coli* BW25113 were grown in LB broth to the late logarithmic phase (OD 600 of ~1) by subculturing (1:100) of overnight cultures into fresh LB for 3 h at 37°C, under aerobic conditions. Total RNA from 18 bacterial cultures (3 species × 2 genetic backgrounds × triplicates) was stabilized by the RNAprotect Bacteria Reagent (QIAGEN) and extracted using the RNeasy mini kit (QIAGEN). Purified RNA was treated with an RNase-free DNase I followed by ethanol precipitation and sequenced by Vertis Biotechnologie AG (Germany). Ribosomal RNA molecules were depleted using a Vertis-developed protocol and fragmented using ultrasound (4 pulses of 30 sec each at 4°C). Oligonucleotide adapters were ligated to the 3ʹ end of the RNA molecules, and first-strand cDNA synthesis was performed using M-MLV reverse transcriptase and the 3ʹ adapter as a primer. Next, the first-strand cDNA was purified, and the 5ʹ Illumina TruSeq sequencing adapter was ligated to the 3ʹ end of the antisense cDNA. The resulting cDNA was PCR-amplified to about 10–20 ng/μl using a high fidelity DNA polymerase, purified using the Agencourt AMPure XP kit (Beckman Coulter Genomics) and analyzed by capillary electrophoresis. The primers used for PCR amplification were designed for TruSeq sequencing according to the Illumina instructions. The libraries pool was analyzed on a Shimadzu MultiNA microchip electrophoresis system and sequenced on an Illumina NextSeq 500 system, generating 259,345,328 single-end reads of 75 bp length. Quality control of the obtained reads using the Illumina tool Sequencing Analysis Viewer showed that over 92% of the reads had quality score of > 30. All RNA-Seq reads were deposited at NCBI in the Sequence Read Archive (SRA) under study number SRP292670, bioproject PRJNA678618, and accession numbers SRR13065767 – SRR13065758 as detailed in Table S4.

**Transcriptome analysis**. The resulting reads were mapped to their correspondent genome with bowtie2 v2.2.9^74^ and reads counts were done with FeatureCounts (version 1.5.0-p1). All statistical analysis was performed using the R (version 3.6.0) software and Bioconductor packages including DESeq2^75^ and the SARTools package developed at PF2 – Institute Pasteur^76^ using the default settings. Normalization and differential analysis were carried out according to the DESeq2 model and package using adjusted *P* value and the Wald test to infer the probability value.

To compare DEGs between *S*. Typhimurium, *S. bongori*, and *E. coli*, we created a dataset consisting of 2,896 orthologous core genes present in all three species (Table S2). Annotated genomes for *S. bongori* serovar 48z41 – strain RKS3044 (accession number NZ_CP006692), *S*. Typhimurium strain SL1344 (accession number NC_016810) and *E. coli* strain BW25113 (accession number NZ_CP009273) were downloaded from PATRIC.^77^ Proteinortho^78^ was used to determine orthology using amino acid fasta files. Phyletic patterns created using the orthology mapping were used to estimate core genomes between three species. Unless otherwise specified, all orthologous genes were named as in *S*. Typhimurium SL1344 to avoid confusion over different annotations of the same gene. Differentially expressed genes (adjusted *P*-value <0.05 and fold change ≥2) of the complete transcriptomes were analyzed by KEGG Mapper,^79^ using the corresponding organism databases. For genes involved in pathways of interest, the normalized reads were plotted in heatmaps using the R pheatmap package (Version 1.0.12).

**Reverse transcription real-time PCR (qRT-PCR)**. Overnight bacterial cultures were subcultured (1:100) into fresh LB broth and grown to the late logarithmic phase under aerobic conditions for 3 h at 37°C. RNA was extracted using the QIAGEN RNA protect bacterial reagent and the RNeasy mini kit, according to the manufacturer’s instructions, including an on-column DNase I digestion. Purified RNA was retreated with DNase I (TURBO DNA-free kit, Invitrogen) according to the manufacturer’s protocol. Up to 1 mg of DNase I-treated RNA was subjected to cDNA synthesis using the qScript cDNA synthesis kit (Quanta-bio). Real-time PCR and data analysis were performed as previously described^80^ on a StepOnePlus Real-Time PCR System (Applied Biosystems). The *rpoD* gene was used as the endogenous normalization control. Fold-differences in gene transcription were calculated as 2^−ΔΔCt^.

**Construction of *S*. Typhimurium His-tagged DksA**. A 468 bp DNA fragment containing the *S*. Typhimurium SL1344 *dksA* gene was amplified by PCR using the primers dksA-NdeI-F and dksA-BamHI-R. The resulting PCR fragment was digested with BamHI and NdeI and cloned into pET-28a digested with the same enzymes, generating the construct pET-28a/*dksA*. This plasmid expresses full-length *S*. Typhimurium DksA fused to a 6× His tag on the N-terminus, with a predicted molecular mass of 18.8 kDa, under the control of the T7 promoter.

**Overexpression of His-tagged DksA and protein purification**. *E. coli* BL-21 (DE3) strain carrying pET-28a/*dksA* was grown overnight in LB medium containing 20 μg/ml Kanamycin at 37°C with vigorous aeration. The culture was diluted 1:15 into 3 ml of fresh LB and grown for 2 h. To induce expression of the recombinant protein, IPTG was added to 1 mM final concentration, and the culture was grown for an additional 4 h at 37°C and harvested by centrifugation. His-tagged DksA was purified using the MagListo His-tagged Protein Purification Kit (Bioneer) according to the manufacturer’s instructions. Protein concentration was measured by the RC DC Protein Assay (Bio-Rad), which indicated 0.8 mg protein/ ml. The purity of the eluted fractions was analyzed on a 15% SDS-polyacrylamide gel (Fig. S1) and stored at −80°C until use.

**Mobility shift assay**. Gel retardation assay was carried out according to.^81^ A 1,100 bp DNA fragment corresponding to the regulatory region upstream from *hilA* was generated using the primers hilA_prom_Fw and hilA_prom_Rev listed in Table S3B. 75 ng of the DNA probe was incubated at 4°C for 60 min with 1 unit of *E. coli* RNAP holoenzyme (New-England Biolabs) and increasing amounts (0.017 to 0.7 µg) of purified His-DksA in the Binding buffer [35 mM Tris-Ac (pH 7.9), 70 mM KAc, 5 mM MgAc_2_, 20 mM NH_4_Ac, 1 mM DTT]. As a negative control for the complex binding, heparin (at a final concentration of 0.15 mg/ml) was added to the binding reaction. Binding reactions were stopped by the addition of 3 µl of loading dye [30% (v/v) glycerol and 0.25% (w/v) bromophenol blue] and immediately resolved on a 4% non-denaturing polyacrylamide gel in 0.5 × Tris-borate running buffer (pH 8.3) at 120 V. The gels were stained with Gelred (Biotium) and recoded by the Fusion Solo X imaging system (Vilber).

**Western blotting**: Bacterial cultures were grown in LB overnight to stationary phase, or diluted 1:100 and grown for additional 3 h to the late-logarithmic phase. The amount of total protein in each culture was measured using the BCA Protein Assay Kit (Pierce) according to manufacturer’s protocol. One ml of each culture was centrifuged, and the pellets were resuspended in 400 µl (for stationary phase cultures) or 100 µl (late log. phase cultures) 1× sodium dodecyl sulfate-polyacrylamide gel electrophoresis (SDS-PAGE) sample buffer. Boiled samples were normalized to equivalent amounts of proteins (12 and 73 µg from stationary and late exponential phase, respectively), separated on 12% SDS-PAGE and transferred to a polyvinylidene fluoride (PVDF) membrane (Bio-Rad Laboratories). Blots were probed with a mouse anti-*E. coli* Sigma S (RpoS) antibody (BLG-663703; BioLegend, diluted 1:1,000). Goat anti-mouse antibody conjugated to horseradish peroxidase (ab6721; Abcam, diluted 1:10,000) was used as a secondary antibody, followed by detection with enhanced chemiluminescence reagents (Amersham Pharmacia). Ponceau-S (Sigma-Aldrich) staining of the membrane confirmed equal amount of proteins loading.

**Motility on soft agar**: Bacterial motility was determined as previously described.^82^ Briefly, overnight bacterial cultures grown in LB broth at 37°C were diluted 1:100 into fresh LB and sub-cultured for 3 h at 37°C with shaking. 10 µL aliquots of the subcultures were placed in the center of 0.3% agar LB plates and incubated for 5 hours at 37°C without being inverted.

**Infection of Bone Marrow Derived Macrophages (BMDMs)**: BMDMs were isolated from 6–8 weeks-old femur of naïve white SWISS female mice. The femur bones were gently separated from the skin and mussels and rinsed with cold PBS supplemented with 1% penicillin-streptomycin. The ends of the bone were truncated and the bone marrow was flashed out using a 27 G syringe needle with cold BMDM medium (49% DMEM high-glucose, 19.5% FBS, 29.5% CSF L-cells condition medium, 1% of 0.2 M L-Glutamate ×100, 1% of 0.1 M pyrovate×100, and 500 µl 0.05 mM β-mercaptoethanol) supplemented with 1% penicillin-streptomycin. Extracted bone marrow was centrifuged at 50 g for 1 min at room temperature, the supernatant was collected and centrifuged at 125 g for 10 min. 5 × 10^6^ cells were plated in non-tissue culture petri dish 145/20 mm prefilled with 20 ml of warm BMDM medium containing 1% penicillin-streptomycin. After 3 days, 10 ml of BMDM medium was added and incubated at 37°C /5% CO_2_ for additional four days. Then the medium was removed, cells were washed with ice-cold PBS without Mg^2+^ and Ca^2+^ (PBS-/-), incubated in 20 ml of ice-cold PBS -/- for 10 min at 4°C and counted.

For the infection and survival assays, 1 ml of BMDM cells (2.5 × 10^5^ cell/ml) were distributed into 24 wells plate for O/N and incubated at 37°C /5% CO_2_. Bacterial cultures were grown in 2 ml LB O/N at 37^°^C on a microbial culture roller drum. On the day of the infection, the O/N culture was diluted to an OD_600_ of 0.2 in a final volume of 1.5 ml of PBS -/-. In order to prepare the bacteria for infection, PBS was replaced with BMDM media and re-diluted 1:50. 100 µl of the diluted bacteria were added into each well. Immediately after the addition of the bacteria, the infected cells were centrifuged for 5 min at 195 g at room temperature to synchronize the infection. After 30 min, the cells were washed 3 times with PBS and replaced with 1 ml of BMDM media containing gentamicin (100 µg/ml). The plate was incubated for one hour at 37°C/5% CO_2_ atmosphere, and then washed three times with PBS that was finally replaced with 1 ml of BMDM media containing 10 µg/ml gentamicin. The plates were incubated until 2 h (T1, for uptake) and 20 h (T2, for survival) post infection.

At the desired time points, BMDMs were washed 3 times with PBS and resuspended in 250 µl of lysis buffer (1% Triton X-100, 0.1% SDS in PBS). The plate was incubated for 10 min under mild shaking. 50 µl of serial dilutions were plated onto LB agar plates and CFUs were counted after overnight incubation at 37°C.

**Quantification of intracellular ppGpp in bacteria**: ppGpp concentration was determined using a liquid chromatography (LC) coupled with a tandem quadrupole mass spectrometer (MS/MS) equipped with an electrospray interface (ESI; operating in positive ionization mode) at the Hannover Medical School Research Core Unit Metabolomics. This method is based on a previously published protocol^83^ with a few modifications. Briefly, overnight bacterial cultures were diluted 1:100 into fresh LB medium and were grown for 3 h. 75 μL of formic acid were added to 2 ml cultures (final formic acid concentration 1 M) and incubated on ice for 1 h. Two mL of ammonium acetate (pH 4.5) was then added and centrifuged at 3000 × *g* for 5 min at 4°C to remove cell debris, while and the supernatants were collected. Solid-phase extraction was conducted using the OASIS WAX 1-cc Vac cartridges (Waters). Each cartridge was pretreated with 1 ml of methanol followed by 1 ml of 50 mM ammonium acetate (pH 4.5). After loading the sample, the columns were washed with 1 ml of 50 mM ammonium acetate and then with 1 ml methanol. Nucleotide pool was eluted with 1 ml of water/methanol/ammonium hydroxide solution, (70/20/10, v/v/v). The effluents were lyophilized in a Speed-Vac at 40°C and kept at −80°C until analysis. Dried samples were dissolved in 200 μL water (HPLC grade) containing 200 nM of ^13^C_10_^15^N_5_-ppGpp as internal standard and analyzed using a LC-ESI-qMS/MS system (QTRAP5500).

Chromatographic separation was carried out on a Shimadzu-Nexera system (Shimadzu) using a Hypercarb-PGC column (30 × 14.6 mm) with a 5 μm particle size (ThermoScientific). A linear gradient of solvent A (10 mM ammonium acetate pH 10) and solvent B (acetonitrile) was applied. The starting conditions were 4% B. Within 8 min, the ratio of solution B was increased to 60% at a flow rate of 600 µL/min. Mass spectrometric detection was performed with a QTRAP5500 tandem quadrupole mass spectrometer (Sciex). All system controls and data analyses were processed by Analyst software (version 1.7; Sciex). ppGpp was detected in positive multiple reaction monitoring (MRM) mode, using mass transition 604 (Q1 mass) to 152 (Q3 mass) for ppGpp. The isotopically labeled ppGpp was used as the internal standard. The detected mass transition was 619 →162.

For quantification, calibration curves were created by plotting peak area ratios of the analyte, and the internal standard versus the nominal concentration of the ten calibrators (range: 0.39–200 pmol/sample). The calibration curve was calculated using quadratic regression and 1/x weighing.

## Supplementary Material

Supplemental Material

## Data Availability

The authors confirm that the data supporting the findings of this study are available within the article and its supplementary materials. The RNA-Seq data was deposited in the Sequence Read Archive (SRA; https://www.ncbi.nlm.nih.gov/sra) under the accession numbers indicated in Table S4.
