# Supplementary material for: The ancestral stringent response potentiator, DksA has been adapted throughout Salmonella evolution to orchestrate the expression of metabolic, motility, and virulence pathways
Source: Gut Microbes. 2021 Dec 20;14(1):1997294. doi: 10.1080/19490976.2021.1997294 (PMC8726615; doi:10.1080/19490976.2021.1997294)
Supplement: Supplemental Material [file KGMI_A_1997294_SM5644.zip › Supplementary information/Supporting information captions.docx]

**Supporting information captions**

**Fig. S1. ppGpp concentration in bacterial cultures grown to late logarithmic phase.** Wildtype and *dksA* isogenic strains of *E. coli*, *S. bongori* and *S*. Typhimurium were grown in LB to the late logarithmic phase and were subjected to ppGpp solid-phase extraction followed by chromatographic analysis on an LC-ESI-qMS/MS system. ppGpp concentration was normalized to protein content, which was determined using a BCA protein assay Kit. The graph shows the mean and standard error of the mean (SEM) of three independent cultures. A *t*-test was used to determine statistical significance of the differences in ppGpp concentrations between strains. ns, not significant.

**Fig. S2. Gene ontology classification of DksA-regulated genes in *E. coli***. Gene ontology analysis was done for 1106 DksA-regulated genes that showed two or more fold change in expression between wildtype and a Δ*dksA* background in *E. coli* BW25113. Upregulated genes are shown in red and downregulated genes in cyan. KEGG pathway classification and the number of the genes grouped in each pathway are shown.

**Fig. S3. Gene ontology classification of DksA-regulated genes in *S. bongori***. Gene ontology analysis was done for 609 DksA-regulated genes that showed two or more fold change in expression between wildtype and a Δ*dksA* background in *S. bongori* NCTC 12419 (SARC 11). Upregulated genes are shown in red and downregulated genes in cyan. KEGG pathway classification and the number of the genes grouped in each pathway are shown.

**Fig. S4. Gene ontology classification of DksA-regulated genes in *S.* Typhimurium**. Gene ontology analysis was done for 917 DksA-regulated genes that showed two or more fold change in expression between wildtype and a Δ*dksA* background in *S.* Typhimurium. Upregulated genes are shown in red and downregulated genes in cyan. KEGG pathway classification and the number of the genes grouped in each pathway are shown.

**Figure S5. DksA regulates motility in an opposite directionality in *E. coli* and *Salmonella*.** Motility of wildtype and *ΔdksA* strains of *S*. Typhimurium (STM), *S. bongori* (SBG) and *E. coli* (EC) were examined on soft agar plates for 5 h at 37°C. The mean motility (distance in cm) of 5 independent cultures and their standard deviation are shown. A student t-test was used to calculate statistical significance.

**Fig. S6. Purification of a His-DksA.** *E. coli* BL-21 (DE3) carrying pET-28a/*dksA* were grown in LB medium supplemented with 20 μg/ml Kanamycin and 1mM IPTG at 37°C. His-tagged DksA was purified from the soluble fraction by nickel-affinity chromatography. The purity of the different fractions is shown following electrophoresis on a 15% SDS-polyacrylamide gel and Coomassie Blue staining.

**Fig. S7. Differences in the expression *ropD* and alternative sigma factors in wildtype and *dksA* mutant.** (**A**) The expression of *rpoD* (σ^70^) and the alternative sigma factors *fliA* (σ^28^), *rpoE* (σ^24^), *rpoH* (σ^32^), *rpoN* (σ^54^) and *rpoS* (σ^38^) is plotted according to their mean number of transcripts per million reads (TPM). The bars show the mean TMP value of three independent RNA-seq analyses of cultures grown in LB to the late logarithmic phase. A *t*-test was used to calculate the statistical significance of the differences between the wildtype and the *dksA* background in *E. coli*, *S. bongori* and *S*. Typhimurium cultures. (**B**) A Western blot using an anti-RpoS antibody was used to detect the expression of σ^38^ in cultures that were grown to the late logarithmic phase (top panel) and to the stationary phase (bottom panel). A Ponceau S staining was used to demonstrate equal loading of total of proteins (73 µg) in each lane.

**List of Tables**

**Table S1.** DksA-regulated genes in *E. coli* (**A**)*, S. bongori* (**B**)*,* and *S.* Typhimurium (**C**) as determined by RNA-seq analyses. (**D**) DksA regulated genes not present in a subset of species

**Table S2**. Core genes regulated by DksA in *E. coli, S. bongori,* and *S.* Typhimurium. (**A**) 2,794 core genes among *S*. Typhimurium, *S. bongori* and *E. coli*. (**B**) Differentially expressed genes among 2,794 core genes.

**Table S3.** Bacterial strains (**A**) and primers (**B**) utilized in this study.

**Table S4.** RNA-Seq deposited at SRA and their accession numbers.
