# Supplementary figures and images for: The ancestral stringent response potentiator, DksA has been adapted throughout Salmonella evolution to orchestrate the expression of metabolic, motility, and virulence pathways
Source: Gut Microbes. 2021 Dec 20;14(1):1997294. doi: 10.1080/19490976.2021.1997294 (PMC8726615; doi:10.1080/19490976.2021.1997294)

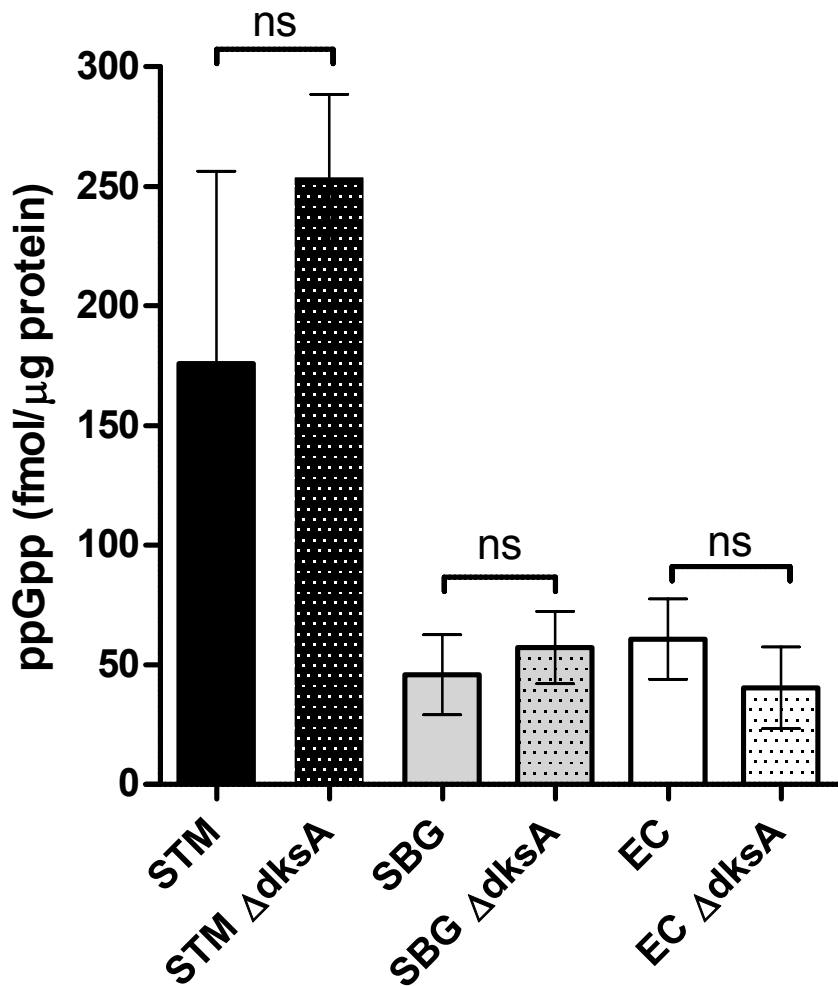

Fig. S1

Supplement: Supplemental Material [file KGMI_A_1997294_SM5644.zip › Supplementary information/Fig S1.pdf]

## *E. coli* pathways

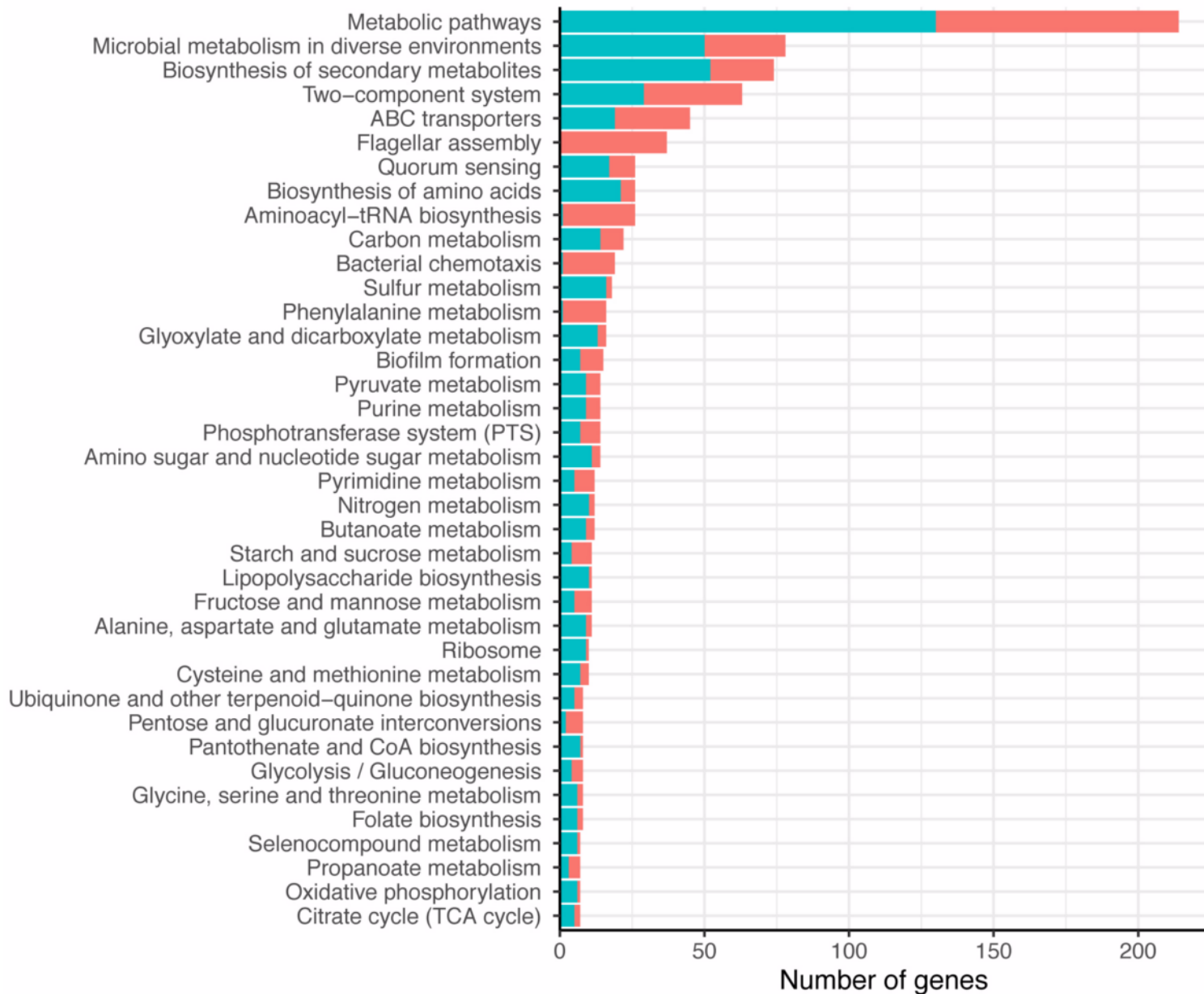

**Fig. S2**

Supplement: Supplemental Material [file KGMI_A_1997294_SM5644.zip › Supplementary information/Fig S2.pdf]

## *S. bongori* pathways

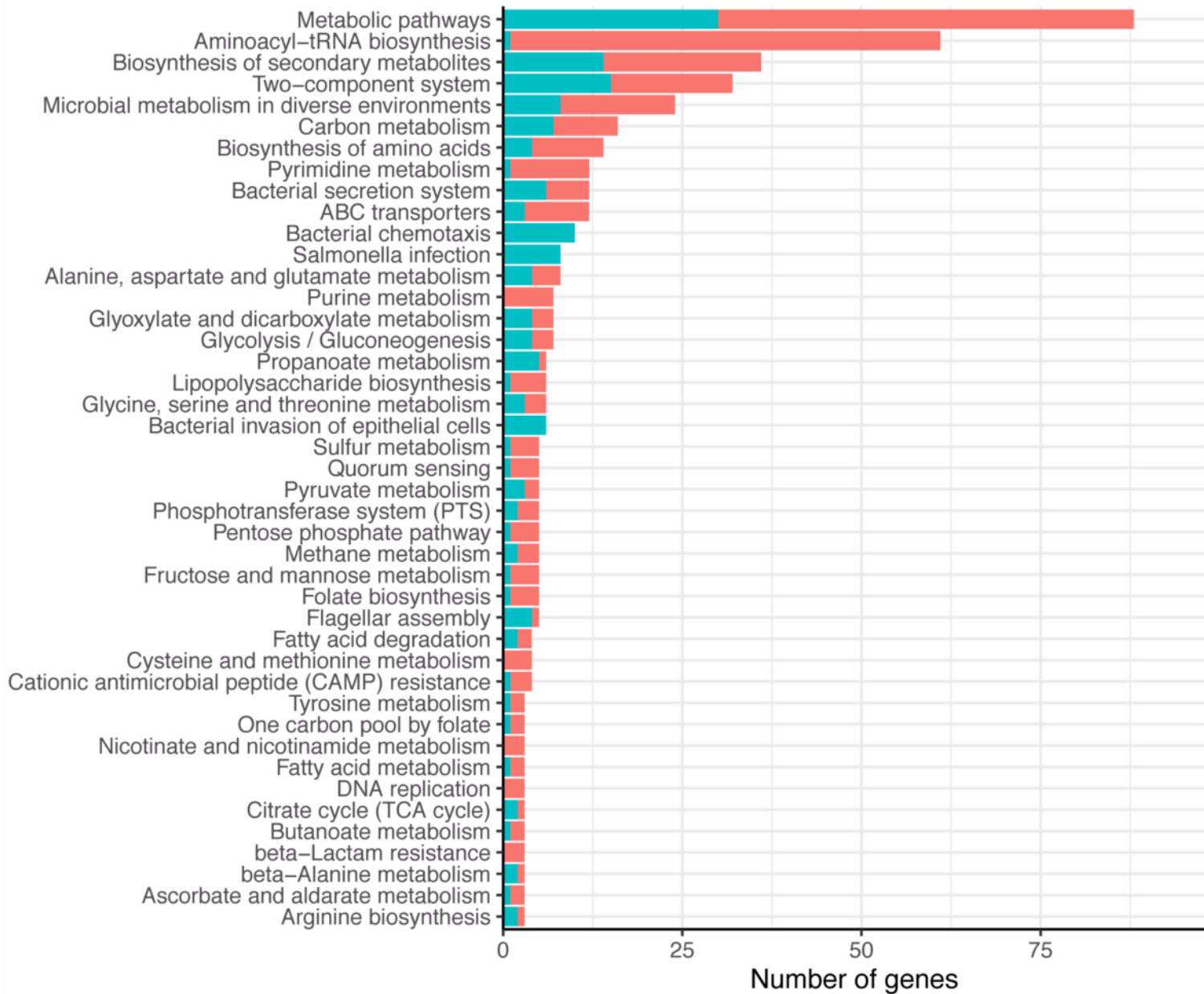

**Fig. S3**

Supplement: Supplemental Material [file KGMI_A_1997294_SM5644.zip › Supplementary information/Fig S3.pdf]

## S. Typhimurium pathways

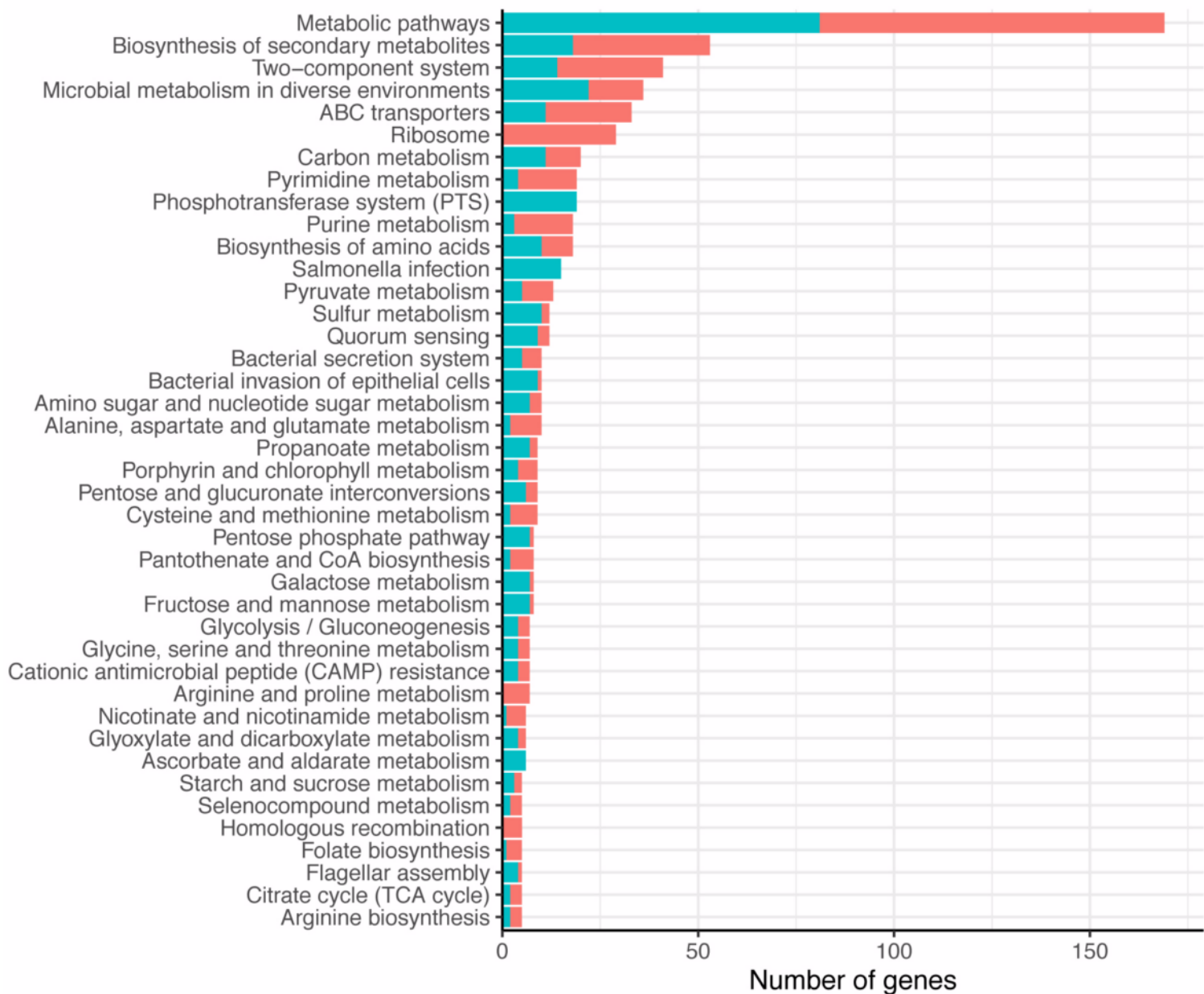

**Fig. S4**

Supplement: Supplemental Material [file KGMI_A_1997294_SM5644.zip › Supplementary information/Fig S4.pdf]

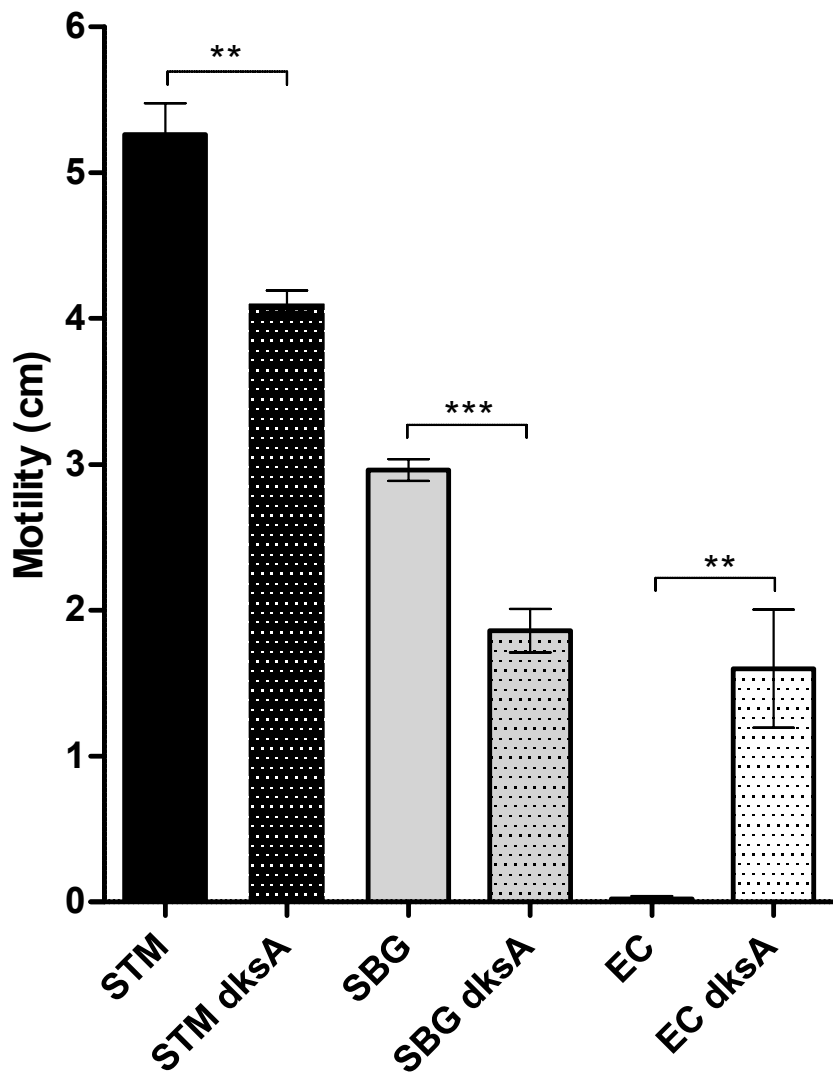

Fig. S5

Supplement: Supplemental Material [file KGMI_A_1997294_SM5644.zip › Supplementary information/Fig S5.pdf]

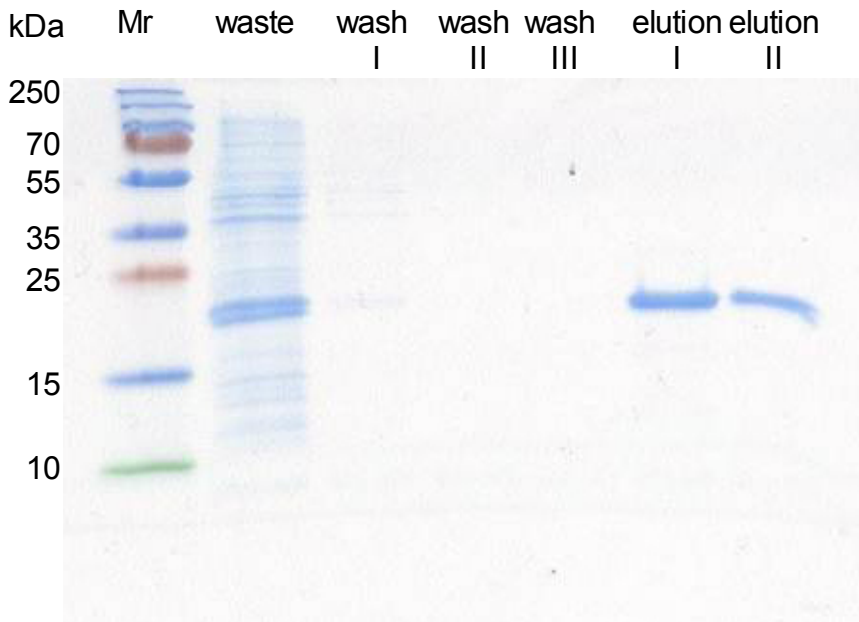

**Fig. S6**

Supplement: Supplemental Material [file KGMI_A_1997294_SM5644.zip › Supplementary information/Fig S6.pdf]

**A**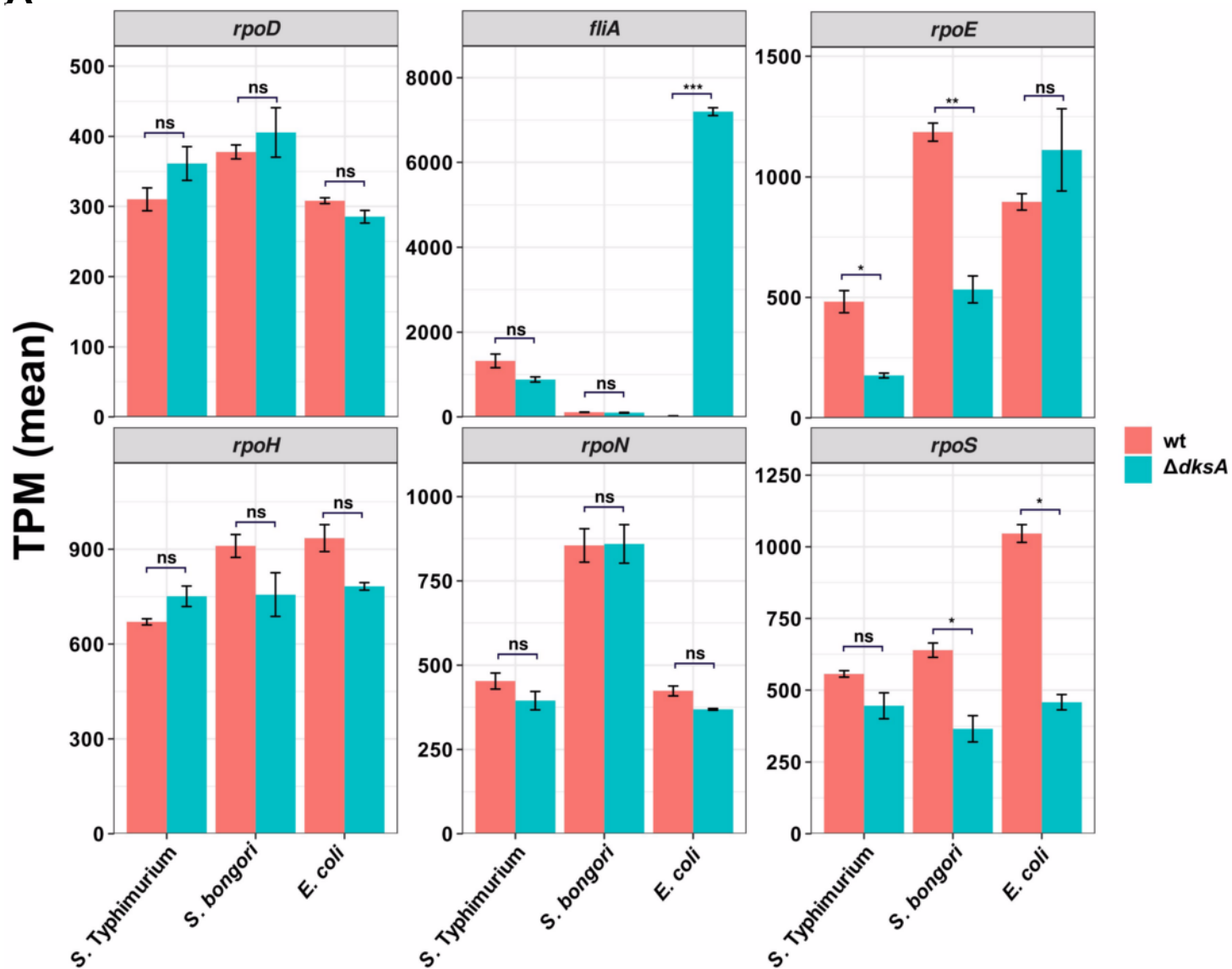**B**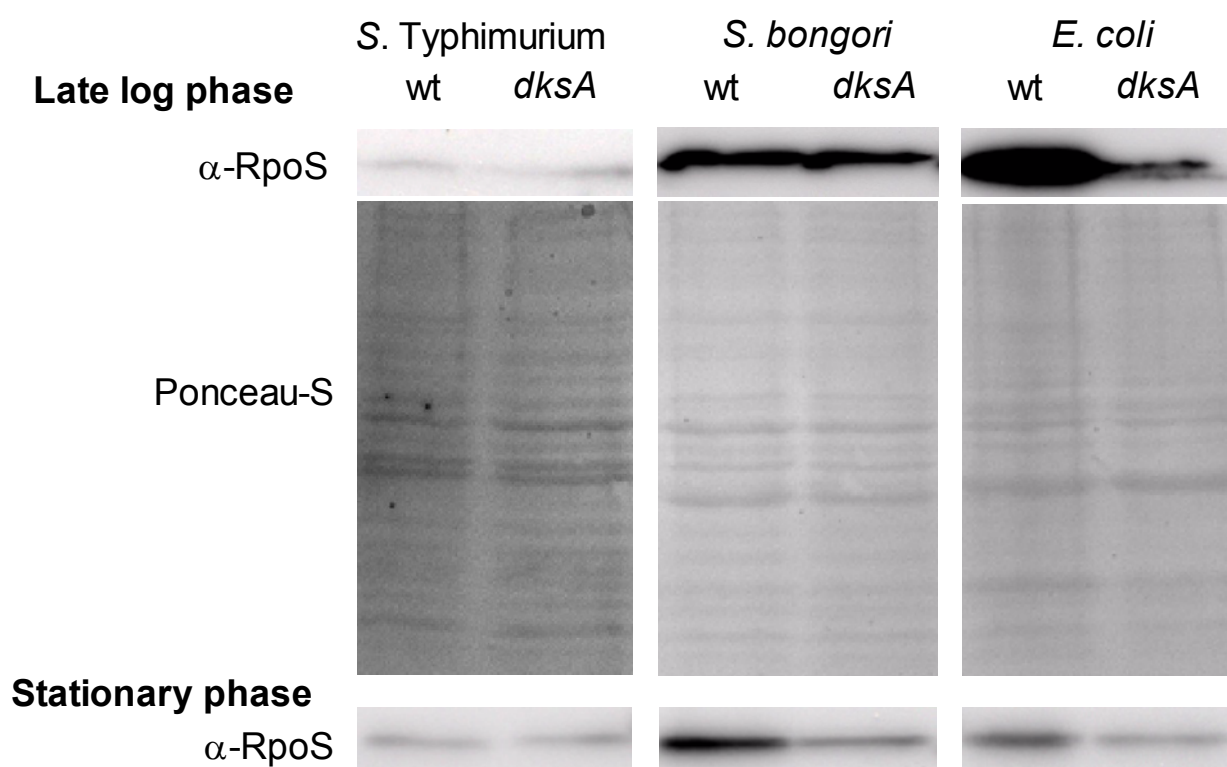**Fig. S7**

Supplement: Supplemental Material [file KGMI_A_1997294_SM5644.zip › Supplementary information/Fig S7.pdf]
